# The role of public health in rare diseases: hemophilia as an example

**DOI:** 10.3389/fpubh.2025.1450625

**Published:** 2025-03-20

**Authors:** Amr A. El-Sayed, Ulrike M. Reiss, Diana Hanna, Nancy S. Bolous

**Affiliations:** ^1^Public Health Institute, Faculty of Health, Liverpool John Moores University, Liverpool, United Kingdom; ^2^Medical Affairs Department, Novo Nordisk Egypt, Cairo, Egypt; ^3^Department of Hematology, St. Jude Children’s Research Hospital, Memphis, TN, United States; ^4^Department of Pediatric Hematology and Oncology, Zagazig University, Zagazig, Egypt; ^5^Phoenix Clinical Research, Middle East and North Africa, Cairo, Egypt; ^6^Department of Global Pediatric Medicine, St. Jude Children’s Research Hospital, Memphis, TN, United States

**Keywords:** public health, rare diseases, hemophilia, orphan drugs, health inequities, public health policy, public health surveillance, disease burden

## Abstract

**Introduction:**

The role of public health has evolved from addressing infectious diseases to encompass non-communicable diseases. Individuals with genetic disorders and rare diseases constitute a particularly vulnerable population, requiring tailored public health policies, practical implementation strategies, and a long-term vision to ensure sustainable support. Given the prolonged duration and significant costs often associated with these conditions, comprehensive, patient-centered, and cost-effective approaches are essential to safeguard their physical and mental well-being.

**Aims:**

To summarize definitions and concepts related to health, public health, rare diseases, and to highlight the role of integrating public health interventions into routine care in improving patient outcomes. Hemophilia was selected as an exemplary rare disease due to its significant lifetime treatment costs and the recent approval and pricing of its gene therapy as the world’s most expensive drug, highlighting the critical importance of public health policies in ensuring equitable access to care and treatment.

**Methods:**

A narrative literature review was conducted between July 2023 and December 2024, searching PubMed, Google Scholar, and Google for various topics related to rare diseases, public health, and hemophilia.

**Results:**

Public health can play an important role in improving the health outcomes of people with rare diseases by implementing conceptual and applied models to accomplish a set of objectives. Over the past two decades, legislative and regulatory support in high income countries (HICs) has facilitated the development and approval of diagnostics and treatments for several rare diseases leading to important advancements. In contrast, many low- and middle-income countries (LMICs) face obstacles in enacting legislation, developing regulations, and implementing policies to support rare disease diagnosis and treatment. More investment and innovation in drug discovery and market access pathways are still needed in both LMICs and HICs. Ensuring the translation of public health policies into regulatory measures, and in turn implementing, and regularly evaluating these measures to assess their effectiveness is crucial. In the case of hemophilia, public health can play a pivotal role.

**Conclusion:**

Enhancing public health surveillance, policies, and interventions in hemophilia and other rare diseases can bridge data gaps, support access to equitable treatment, promote evidence-based care, and improve outcomes across the socioeconomic spectrum.

## Introduction

Individuals with genetic disorders and rare diseases constitute a particularly vulnerable population, given the prolonged duration and significant costs often associated with these conditions (1–4). Thus, tailoring public health policies, executing practical implementation strategies, and developing long-term plans to ensure sustainable support can contribute to alleviating the humanistic and economic burden associated with these inherited conditions (5–8). To accomplish that, the definition of health needs to embrace this vulnerable population, whose health is shaped by their unique genetic characteristics, which negatively impact their quality of life and well-being (9, 10). Therefore, providing optimal care for these individuals to address their health problems will enable them to cope with their health condition and to experience and enjoy a sense of health and well-being (11). Hemophilia is the most common inherited bleeding disorder, affecting more than 273,000 people, with an estimated additional 563,000 undiagnosed people worldwide (12, 13). Hemophilia was chosen as an exemplary rare disease due to its substantial lifetime treatment costs and the severe health consequences of inadequate management (14–17). Furthermore, hemophilia stands out as one of the few rare diseases with an approved gene therapy, currently recognized as the most expensive drug in the world (18–20). As such, public health policies play a critical role in ensuring equitable access to care and novel treatments (21–24).

This narrative review aims to summarize and discuss various aspects related to concepts of health, public health, rare diseases in general, and hemophilia in particular, and highlight that the integration of public health interventions into routine care may improve the outcomes for patients affected by rare diseases, including hemophilia.

## Methods

We embarked on an in-depth narrative literature review to explore and discuss the role of public health in rare diseases in general, using hemophilia disease as an example. First, we conducted a preliminary search of the literature, which yielded a range of heterogenous sources, mostly narrative literature reviews, and highlighted various sub-topics that we believed added value to the main topic. Due to this heterogeneity, a systematic search for primary studies seemed inapplicable, and it was deemed necessary to employ a narrative review methodology because it was more appropriate for our broad topic with its multifaceted aspects (25, 26). Accordingly, we conducted focused and snow-balling searches between July 2023 and December 2024, capitalizing on basic and advanced search techniques on PubMed, Google Scholar, and Google (27, 28). We used several keywords and phrases related to rare diseases, public health, and hemophilia to identify relevant publications in the English language, with no date limits or selective geographical locations. The search terms used were basic definitions, epidemiological aspects, economic burden, psychosocial burden, legislations, regulations, policies, implementation gaps, access to treatment, and other secondary topics. Relevant concepts and themes identified during the search process were then classified and described under headings and sub-headings in this review (25, 26).

## Results

### Search results

**Figure 1:**
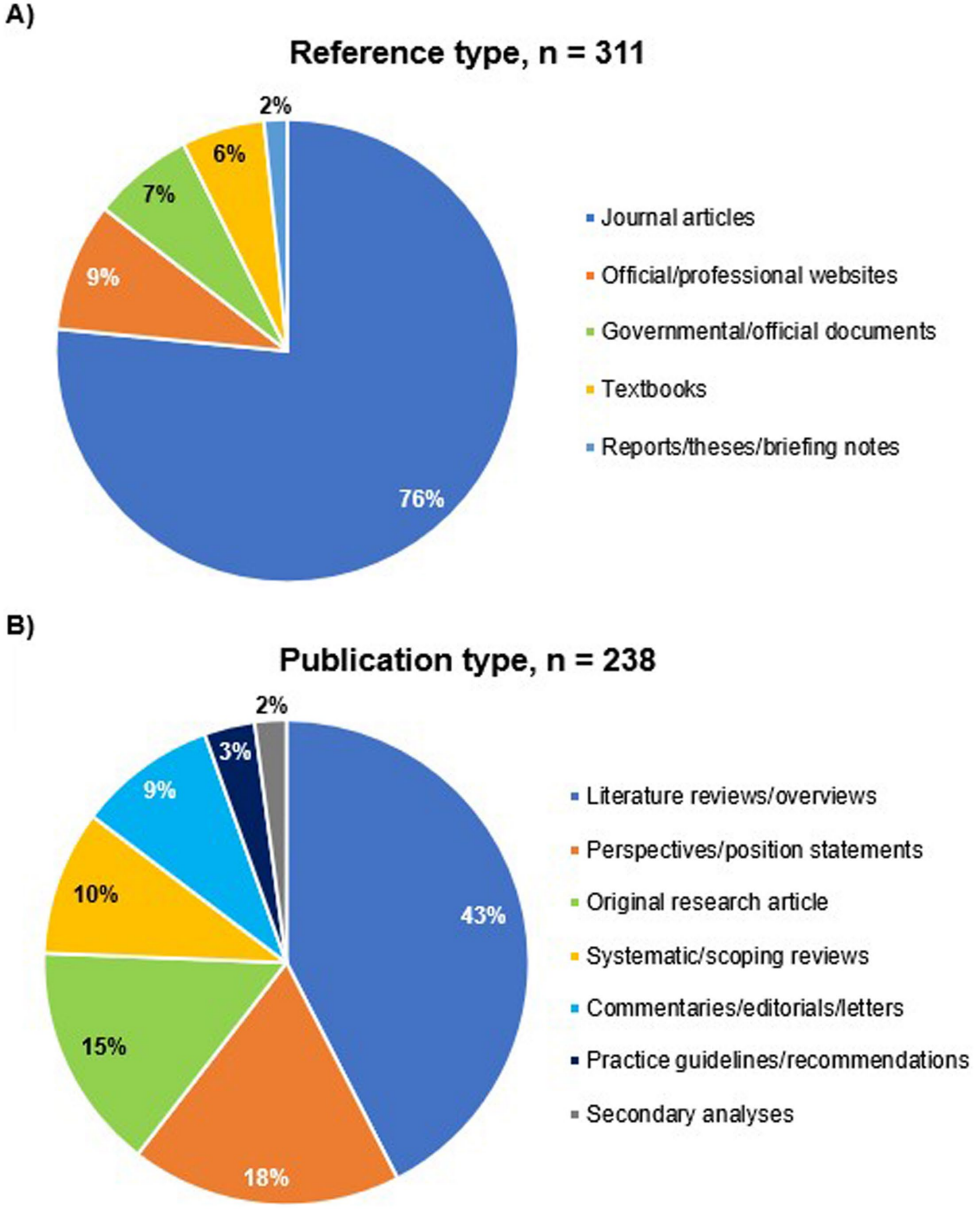
Classification of sources of evidence used to synthesize information in this review. **(A)** Topic-related references, **(B)** journal articles.

Our online searches identified 315 relevant sources of evidence, which were used to synthesize information spanning various topics covered in this review (Supplementary Table 1). After that, we classified the retrieved sources according to (1) relevance (topic-related or methodological references), (2) Publication type (journal articles, textbooks, governmental/official sources, websites, etc.), (3) type of evidence (original research articles, literature/systematic reviews, online books/book chapters, practice guidelines/recommendations, governmental/official reports/papers/guidance, etc.), and (4) evidence category (primary or secondary source of evidence). The topic-related sources were 311 references, of which journal articles represented 76.5% (238) of sources (Figure 1A). Among these 238 journal articles, 179 (75.2%) were narrative reviews and articles, while 36 (15.1%) were original research articles and 23 (9.7%) were systematic and scoping reviews (Figure 1B).

### Relevant definitions and concepts

#### Definition of health

The original definition of health set by the World Health Organization (WHO) in 1946 necessitated the absence of disease or infirmity to achieve the actual meaning of health (29). However, a contemporary and more dynamic definition of health argues that health and disease or disability may co-exist without prejudicing the value of health (30). Thus, people with chronic diseases and disabilities can enjoy healthy lives if they receive appropriate medical care and are able to cope with their condition (11). As a result, new definitions for health have been proposed, underscoring the dynamic balance among the structural, functional, physical, mental, social, and emotional states of the individual in adapting to life and the environmental conditions to attain an effective state of personal well-being as part of the society (31, 32). Nonetheless, these new definitions may not be suitable for all health conditions due to the complexity of the health notion across various conditions and different stages of life, where both illness and well-being are dynamic and interwoven states. Therefore, health can be attained when a person can cope with these various health states involved in defining the overall health condition (9, 10). Furthermore, the increasing use of technology and the digitalization of healthcare make the adoption of a single definition of health more complex (33).

#### Definition of public health

Public health can be defined as the science and practice of protecting, promoting, and maintaining good health and quality of life, as well as prolonging the lives of all people (34–36). This can be achieved by detecting, preventing, and managing disorders, diseases, illnesses, and injuries through organized public health measures and actions taken by public and private institutions, non-governmental community-based organizations, and individuals (37–39). Therefore, public health constitutes an integral part of the healthcare system (9). While clinical healthcare focuses on treating individuals or subgroups of people in times of sickness, public health focuses on protecting and promoting the health and well-being of the entire population to meet the growing needs and expectations of society (9, 34). This evolving role of public health has led to developing the more recent concept of population health. This term, which can be used as a synonym for public health, emerged to address and improve health outcomes and their distribution among all community members over time, by considering broader factors that influence these outcomes. These factors encompass demographic and socioeconomic variables that contribute to health inequities in the community (40–42).

#### Conceptual framework for public health practice

**Figure 2:**
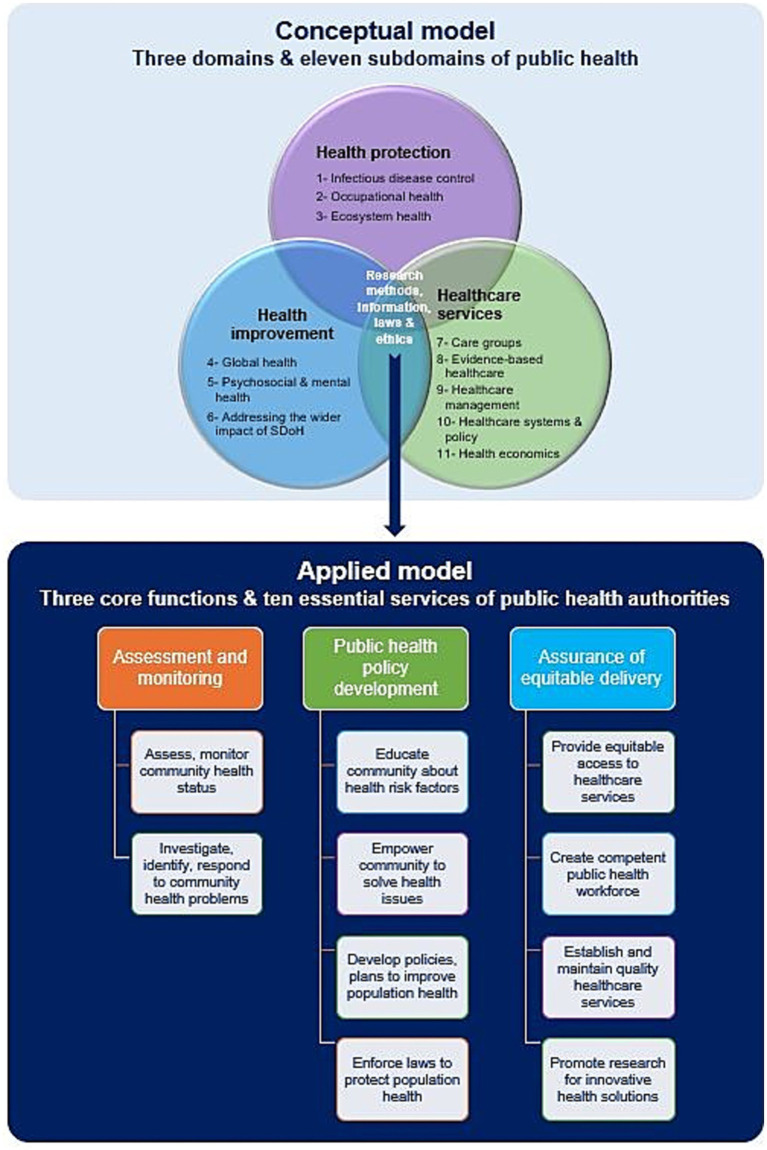
Conceptual and applied models of public health practice. Data modified from references (39, 44, 46). SDoH, Social determinants of health.

Figure 2 summarizes the conceptual and applied models of public health practice. The former consists of three overlapping main domains and 11 subdomains. The area of overlap represents the role and activities of public health directories, capitalizing on the use of research methods, information technology, laws, and ethics in public health practice. This conceptual framework establishes a culture of public health assessment that relies on surveillance and monitoring of health hazards and adopts governance and risk management strategies using public health intelligence and information technology (37, 39).

The applied model of public health practice is based on the following three core functions of public health authorities and agencies: (1) public health surveillance through assessment and monitoring of health information of populations at risk, (2) development of comprehensive public health policies to tackle the identified health problems and set priorities for their management, and (3) assurance that appropriate and cost-effective public health services are provided to the community equitably. These three core functions can be implemented by public health entities by delivering the 10 essential public health services (9, 43–46).

#### Definition of determinants of health and health inequities

Determinants of health and health inequities are the social, economic, environmental, and political factors that predict the individual’s health, such as education, income, housing, unemployment, nutritional status, living and working conditions, psychosocial support, and ways of transport, which in turn, are influenced by public policies. Those health-shaping factors mostly exist outside the healthcare system, which itself remains one of the social determinants of health (47–50). We have identified 50 determinants of health in the scientific literature and official websites of main public health authorities, with additional determinants specific to certain diseases and disorders that may be added to the list (Supplementary Table 2) (47, 51–55). These determinants of health are broadly classified into three main categories: the individual’s characteristics and behaviors, the individual’s physical environment, and the individual’s social and economic environment (56, 57). Differences in those factors among society members create avoidable and unjust inequities in health and well-being outcomes (49, 58–61). In addition, biological and genetic characteristics are at the core of several interrelated factors that affect the individual’s health and well-being (55, 62).

#### Definition of rare diseases and orphan drugs

Despite the growing international recognition of rare diseases, there is no consensus on a unified global definition for rare diseases (63–73). A systematic search conducted between 2013 and 2014 identified 296 different definitions for rare diseases from 1,109 entities in 32 countries (67). The prevalence threshold for rare diseases ranged from 5 to 76 cases per 100,000 population. Moreover, 21 out of the 32 countries (66%) adopted a prevalence threshold from 40 to 50 cases per 100,000 population. The overall prevalence threshold average was 40–50 cases per 100,000 population (67).

Table 1 summarizes different definitions of rare diseases proposed by various countries and jurisdictions, as an integral part of their public health policies designed for rare diseases, which will be discussed later. The European Union (EU) defines a rare disease as a severely debilitating and life-threatening or seriously progressive and chronic disorder affecting ≤50 per 100,000 people (74) which requires medical attention to reduce its morbidity and mortality, and its impact on the person’s quality of life and social integration (72, 75, 76). In 2019, Health Canada adopted a definition and a prevalence threshold similar to the EU (77, 78).

**Table 1:** Definition and prevalence threshold for rare diseases across several countries, regions, and agencies.

Country/region/agency	Definition of a rare disease	Year of proposing the definition	Prevalence threshold
WHO (63, 79–81)	A disease affecting <6.5–<10 per 10,000 persons	Not reported	<65–<100 per 100,000 people
United States (64, 84–86, 190, 192)	A disease affecting <200,000 persons	1984	<60 per 100,000 people (in 2023)
Singapore (63, 65, 70, 94)	Severely debilitating, and life-threatening disease affecting <20,000 persons	1991	<34 per 100,000 people (in 2023)
Japan (63, 66, 79, 89–93, 313)	A disease of unknown etiology with no effective treatment and affecting <50,000 persons	1995	<40 per 100,000 people
European Union (74)	Severely debilitating, and life-threatening, or seriously progressive, and chronic disorder affecting ≤5 per 10,000 persons	1999	≤50 per 100,000 people
Taiwan (65, 90, 105)	A disease affecting <1 per 10,000 persons, has a genetic origin, and is difficult to diagnose and treat	2000	<10 per 100,000 people
South Korea (65, 90, 95, 99)	A disease affecting <20,000 persons or with unknown prevalence due to unavailability of diagnostic tools or appropriate treatment	2003	<39 per 100,000 people (in 2023)
Colombia (65, 73, 101, 290, 314)	A disease affecting ≤2 per 10,000 persons	2010	≤20 per 100,000 people
Argentina (65, 73, 101, 314)	A disease affecting ≤5 per 10,000 persons	2011	EU threshold
Peru (65, 73, 101, 106)	A disease affecting ≤1 per 100,000 persons	2011	≤1 per 100,000 people
Russia (70, 104, 106)	A disease affecting ≤1 per 10,000 persons	2011	≤10 per 100,000 people
Mexico (65, 73, 101, 314)	A disease affecting ≤5 per 10,000 persons	2012	EU threshold
Brazil (65, 73, 101, 289, 290, 314)	A disease affecting ≤6.5 per 10,000 persons	2014	≤65 per 100,000 people
Chile (65, 73, 101)	A disease affecting ≤5 per 10,000 persons	2015	EU threshold
Philippines (65)	A disease affecting ≤0.5 per 10,000 persons	2016	≤5 per 100,000 people
Australia (70, 102)	EU definition	2017	EU threshold
Canada (78)	EU definition	2019	EU threshold
India (96–98)	A disease affecting ≤500,000 persons	2019	≤20 per 100,000 people
China (268, 312, 315)	A disease affecting <1 per 10,000 persons, incidence at birth <1 per 10,000 newborns, and/or <140,000 persons	2021	<10 per 100,000 people
Türkiye (100)	EU definition	2022	EU threshold

EU, European Union; WHO, World Health Organization.

The WHO set a threshold for rare disease prevalence of less than 65 to less than 100 per 100,000 population (63, 79–81). The United States (US) considers a disease rare when it affects <200,000 persons nationally, corresponding to 85 per 100,000 people in 1984 when the Orphan Drug Act was amended. As the US population grew, the prevalence declined over the years until it reached <60 per 100,000 population in 2023 (64, 70, 82–87). The change in the prevalence of rare diseases in the US pertains to the arbitrary absolute threshold of less than 200,000 persons nationally definition, which was derived from previous estimates on narcolepsy, multiple sclerosis, and Tourette syndrome (70, 71, 87, 88). Similarly, Japan adopted an absolute figure approach and identified a prevalence threshold below 50,000 persons in 1993 for each rare disease (63, 66, 79, 89, 90). The prevalence at that time was equal to approximately 40 per 100,000 persons, which was almost the same in 2023 with comparable population size (91–93). Additionally, other countries such as India, Singapore, and South Korea followed a similar approach to that of the US and Japan (63, 65, 70, 90, 94–99).

While some other countries, such as Argentina, Australia, Chile, Mexico, and Türkiye adopted the EU definition and prevalence threshold for rare diseases (65, 70, 73, 78, 100–102), others, such as Colombia, Peru, Philippines, Russia, and Taiwan adopted stricter prevalence thresholds (65, 70, 73, 90, 101, 103–106). In China in 2021, a rare disease definition included a prevalence threshold of <10 per 100,000 persons, incidence at birth of <10 per 100,000 newborns, and/or a total affected population of <140,000 persons nationwide (107).

According to the most widely accepted prevalence thresholds for defining rare diseases worldwide, hemophilia and other inherited bleeding disorders are thus considered rare (79, 108).

Drugs that treat rare diseases are considered orphan medicinal products by the EU, the Food and Drug Administration (FDA), and other regulatory bodies if the available treatment options are unsatisfactory or do not currently exist or may not exist in the future (106).

### Burden of rare diseases

Disease burden is a term used to estimate the magnitude of a disease or health condition and its impact on a target population by collecting and reporting morbidity and mortality measures (109, 110). Morbidity measures of disease frequency include incidence rates and prevalence proportions (111, 112). The lost healthy life years, calculated as disability-adjusted life years, can be used as a composite outcome measure of the consequences caused by a disease’s morbidity and mortality (113, 114). In addition, estimating the disease burden by considering the economic aspects of disease management and its complications is called cost-of-illness (115, 116). Ideally, it should consider all direct and indirect costs spent by healthcare and non-healthcare sectors in society, as well as productivity loss by patients and caregivers due to the disease (117–119).

#### Prevalence of rare diseases

Recent estimates show that the number of currently identified rare diseases exceeds 10,390 (120), of which around 80% have a genetic origin and up to 75% have a pediatric onset (72, 79). It was estimated that at least 5.9% of the health conditions affecting humans are caused by 3,585 rare disorders, corresponding to a minimum of 446 million people globally living with rare disorders from 2017 onwards. However, only 4.2% of rare diseases are responsible for up to 80.7% of the rare disease burden. These rare diseases have a prevalence of 1–5 per 100,000 people. On the other hand, 84.5% of rare diseases have a prevalence of <1 per 1,000,000 people (71).

In the year 2000, the number of people living with a rare disease in the 25 countries constituting the EU was approximately 225,000 persons within a population of 450.4 million inhabitants (121). This number remained almost the same in 2023 based on a population of 448.4 million inhabitants in 27 countries (122).

Hemophilia affected more than 273,043 people worldwide in 2023 (13). The prevalence of hemophilia was extrapolated from national patient registries in six high-income countries. Data extrapolations estimate an additional 563,000 undiagnosed people with hemophilia worldwide. Of the total 836,000 diagnosed and undiagnosed patients, approximately 284,000 individuals are expected to be severe cases, based on a world population of 8 billion in 2023 (13, 123). Hemophilia severity is defined according to baseline factor levels (severe <1%, moderate 1–5%, mild >5–<40%) (124, 125) and clinically correlates with the number of bleeding episodes per year (126, 127). Without implementing prophylaxis as a standard of care, severe hemophilia is associated with shorter life expectancy, higher rates of musculoskeletal complications, and reduced quality of life and well-being (128, 129).

#### Psychosocial burden of rare diseases

Rare diseases significantly reduce health-related quality of life and mental health, leading to negative psychosocial and emotional impacts on both patients and caregivers (130). These negative consequences are caused by stigma in school and workplace, including social exclusion, misappreciation, discrimination, lack of social support or understanding, and bullying from peers and teachers (131).

A recent study found that more than 75% of caregivers of 41 children and adolescents living with rare diseases in Western Australia experienced stigma, and over 46% reported being bullied at school (132). Several factors contribute to these negative behaviors, including a lack of or poor understanding of the rare condition and its complications among non-specialized healthcare professionals and the public, delayed or misdiagnosis of the disease, inadequate medical care—including psychosocial support, and a heavy reliance on caregivers, particularly mothers, lack or unavailability of effective treatment options, loss or reduced productivity, high out-of-pocket expenditure on medical care, and difficulties managing administrative tasks and socio-legal issues to receive the appropriate care they deserve (133–137). These challenges are more prominent in low- and middle-income countries (LMICs), where diagnostic and therapeutic options are limited due to scarce resources allocated for health systems, especially for people with rare diseases (138).

In hemophilia, the psychosocial burden experienced by patients is influenced by musculoskeletal health, which can be affected by the number of target joints, joint disabilities, the duration on episodic treatment or non-optimal prophylaxis, especially during childhood (15, 128, 139).

#### Economic burden of rare diseases

Rare diseases are associated with a considerable economic burden to both the healthcare sector and society (105, 140–143). In an economic analysis that included 24 rare diseases from five disease categories in the USA, the annual total economic burden per patient was approximately 10 times higher than that of other chronic diseases, such as diabetes and cardiovascular disorders (8). In Hong Kong in 2021, the average annual total cost per person across 106 rare diseases was reported to be 62,084 US$. The total out-of-pocket healthcare expenditure was estimated at 6,646 US$ per patient per year, representing approximately 11% of the total cost. Out-of-pocket expenditure on healthcare exceeded 10% of the total household income in more than 36% of families with a person affected by a rare disease. This catastrophic expenditure on healthcare pushed approximately 9% of families below the poverty line (141). In the US, out-of-pocket expenditure on healthcare for rare diseases was 4% of the total cost (143). Moreover, direct non-medical and indirect costs accounted for 51% and 61% of the total cost of rare diseases in the US and Hong Kong, respectively (141, 143).

Among 83 rare diseases in Sichuan province in China, hemophilia was associated with the highest total cost of care (144). A recent scoping review found that the annual societal cost of severe hemophilia A and B without inhibitor across 14 countries ranged from 479 US$ in India to 700,070 US$ in the US, with clotting factor replacement therapy accounting for 95.1%–99.9% of the total cost. In cases of inhibitor development, the annual cost was five to seven times higher than that for severe patients without inhibitors with a reported range of 1,289,663 US$ to 1,780,903 US$ (14). Other studies found that the annual cost of hemophilia A and B without inhibitors ranged from 201,471 US$ to 621,273 US$ in the USA (145, 146) and from 199,541€ to 246,693€ in Europe (147, 148), with significant out-of-pocket expenditure on hemophilia care in LMICs (149, 150).

### The three core functions of public health

#### Public health surveillance

Surveillance is an essential function of public health services, defined as the continuous and systematic collection, analysis, interpretation, and dissemination of health data needed for planning, implementing, and evaluating public health activities. These health-related data are collected from various sources, including patient organizations and official healthcare system registers. Without sufficient and systematic data collection, a surveillance system cannot function properly (151–153). It involves the timely dissemination of information to those responsible for disease prevention and control, as well as to those who require the data to take appropriate action (154–158). Additional aspects of the surveillance system include disease management by providing the required diagnostic and clinical services; training and education of the healthcare staff; information management systems that support data collection, data analysis, and reporting of findings; as well as policy formulation and enactment to support the implementation of surveillance (155, 156, 159).

After data collection, the next step is analyzing and interpreting the information using a cross-sectional study design to characterize the target population and to identify potential risk factors, as well as disease and treatment outcomes of interest, which can be further analyzed for predicting and monitoring disease trends over time (160, 161). The final step in public health surveillance is sharing the findings of these analyses and interpretations with those responsible for designing and implementing better health policies, allocating sufficient healthcare resources, and finally improving patient access to available treatment options (154–156, 161).

In hemophilia, surveillance plays a critical role in detecting and monitoring incidence rates, prevalence proportions, and mortality rates (162–164). It is also helpful in collecting and mapping individual and disease characteristics in the affected population, including identifying risk factors for developing subsequent serious and life-threatening disease complications, such as intracranial hemorrhage, inhibitor development, musculoskeletal complications, and other co-morbid conditions, such as blood-borne infections and cardiovascular disease in the adult population (161, 165, 166). Table 2 provides an overview of public health surveillance systems and registries for hemophilia and inherited bleeding disorders (13, 161, 162, 167–174).

**Table 2:** Hemophilia and inherited bleeding disorders public health surveillance systems and registries.

Surveillance system/registry	Period of data collection	Duration of data collection	Country/region	Type of data collected
Hemophilia Surveillance System (HSS) (161, 172)	From 1993 to 1998	6 years	6 states in the US	Incidence rate, sources of provided care, as well as disease and treatment complications and outcomes
US Universal Data Collection system (UDC) (161, 162, 172)	From 1998 to 2011	14 years	129 HTCs across the US	Transfusion-transmitted infections and joint arthropathy
Community Counts (162, 169, 172)	From 2011 and onwards	15+ years	140 HTCs across the US	Disease and treatment complications and outcomes
European Haemophilia Safety Surveillance (EUHASS) (171, 173, 174)	From 2008 and onwards	18+ years	99 HTCs in 27 European countries	Adverse events related information, unexpected ineffectiveness, neoplasms, and mortality
WFH annual global survey (13, 161, 168, 174)	From 1998 and onwards	28+ years	147 countries worldwide	Demographic, epidemiological and treatment-related information
WFH World Bleeding Disorders Registry (WBDR) (170)	From 2018 and onwards	8+ years	87 HTCs in 40 countries	Sociodemographic, diagnostic, and clinical information collected at baseline and regular follow-up visits
PedNet Haemophilia Registry (167, 174)	From 2004 and onwards	22+ years	33 HTCs in 19 countries	Diagnostic, clinical, and treatment-related information collected at baseline and regular follow-up visits
Country-specific registries (174)	From 2009 and onwards	17+ years	China for example	Demographic, epidemiological, diagnostic, and clinical information, based on data availability of the country

HTCs, Hemophilia Treatment Centers; PedNet, Paediatric Network on haemophilia management; US, United States of America; WFH, World Federation of Hemophilia.

Patient registries play a pivotal role in addressing the gaps in epidemiological data for hemophilia and other rare diseases as crucial sources of information for basic and clinical research, as well as for epidemiological and public health purposes (174, 175). It is evident that national and international registries support collecting standardized data, improving data quality, and enhancing our understanding of the disease’s epidemiology for better public health planning (98, 176). These registries provide comprehensive data on patient demographics, disease characteristics, treatment patterns, and outcomes (151, 170, 177). Notably, long-term population registries could improve methods for data collection, enhance the accuracy of estimating epidemiological data, and support informed decision-making in managing hemophilia and rare diseases (68, 175).

#### Public health policies

Public health policy is a broad term that refers to official laws, regulations, procedures, measures, actions, decisions, plans, and incentives designed by governments, as well as relevant authorities and institutions, to promote the health and well-being of a target population and to ensure achieving specific health goals for that population group (178–180).

In 2015, with the adoption of the 2030 agenda by the United Nations General Assembly (UNGA), one of the targets of sustainable development goal number three (SDG 3) was universal and equitable health coverage for all people without any kind of distinction or financial burden (181). The role of public health has evolved to include individuals living with rare diseases (3). This was accomplished through enacting legislative actions, enforcing regulatory measures, and designing and implementing national plans, frameworks, policies, and strategies (65, 75, 182–185). During the last two decades of the 20^th^ century, various stakeholders, including legislative bodies, regulatory authorities, research institutions, and other governmental and non-governmental entities in several countries started to realize the need for people living with rare diseases to have effective treatments for their lifelong conditions and to support them. This was done by releasing decrees and adopting regulations to incentivize research institutions and the pharmaceutical industry to develop treatment options for various rare diseases (63–66, 68, 70, 75, 76, 79, 182, 184–189).

**Figure 3:**
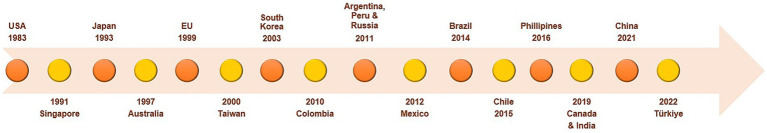
Timeline of legislations, regulations, and national policies for rare diseases and orphan drugs in different countries. Data summarized from references (64–66, 70, 73, 74, 78, 89, 90, 95–97, 99–102, 104, 105, 192, 268, 289, 312). EU, European Union; USA, United States of America.

**Figure 4:**
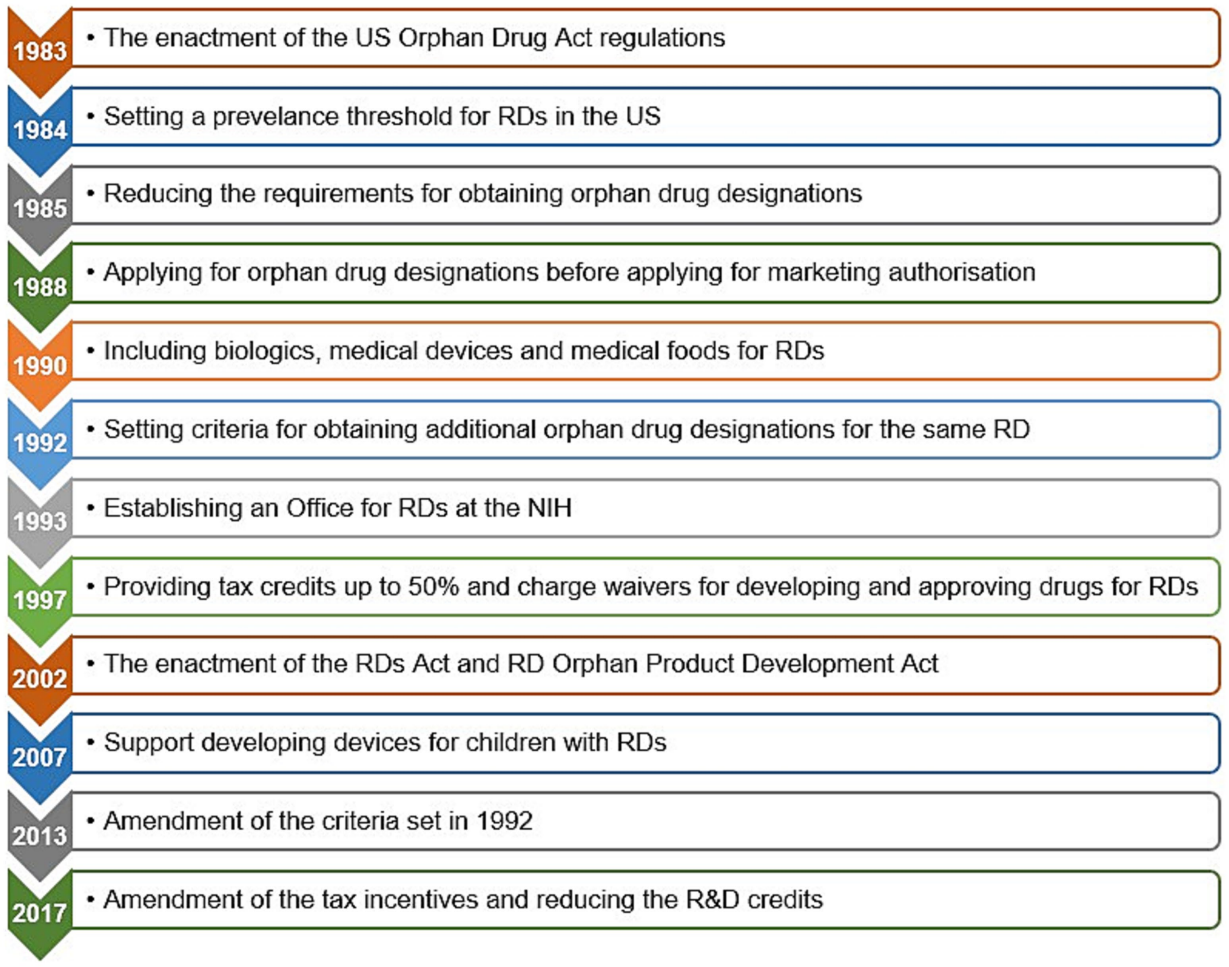
The Orphan Drug Act and subsequent amendments in the United States of America. Data summarized from references (64, 84–86, 190, 192). NIH, National Institutes of Health; RD(s), rare disease(s); R&D, research and development; US, United States.

Figures 3, 4 present the timelines of key legislations, regulations, and national policies related to rare diseases and orphan drugs in the US and globally. A notable example is the US Orphan Drug Act, which was enacted in 1983 and subsequently amended several times, with the latest amendment in 2017 (64, 70, 85, 86, 190–192) (Figure 4).

Since the enactment of these legislative measures and regulatory instruments, rare diseases have been given attention as a global public health priority in health policy, medical research, and regulatory agendas, which in turn reflected positively on orphan drug pipelines (68, 71, 72, 76, 81, 185, 193–195). During the period between the enactment of the Orphan Drug Act by the US Congress in 1983 until the end of 2022, the US FDA granted 6,340 orphan drug designations for 1,079 rare diseases, of which 882 orphan drugs, representing 14% were approved for 392 rare diseases.

Similarly, since the enforcement of the European regulation on orphan medicinal products in 2000, the European Commission designated around 2,000 therapeutic agents as orphan medicinal products and approved 200 of them (74, 196). Furthermore, in 2021 and 2022, 52% and 49% of new drug approvals, respectively, were assigned to rare diseases (197, 198).

#### Public health role in the provision of cost-effective, accessible and equitable care

Stemming from the overarching principle of providing appropriate healthcare services for all people (199), the UNGA recognized the needs and challenges faced by people living with rare diseases. In December 2021, a complementary resolution was adopted to focus on this specific population and their families (200). The resolution aims to ensure that they can exercise their human rights to achieve the highest level of physical and mental health, as well as to promote their inclusion and participation in society (201).

The International Rare Disease Research Consortium was founded in 2011 with an ambitious goal of discovering diagnostic tools for most rare diseases by 2020 and getting 1,000 new therapies approved for rare diseases by 2027. The first goal was achieved earlier than expected in early 2017 due to the allied global efforts for serving the rare disease community, whereas the second goal is still underway (202–204).

**Figure 5:**
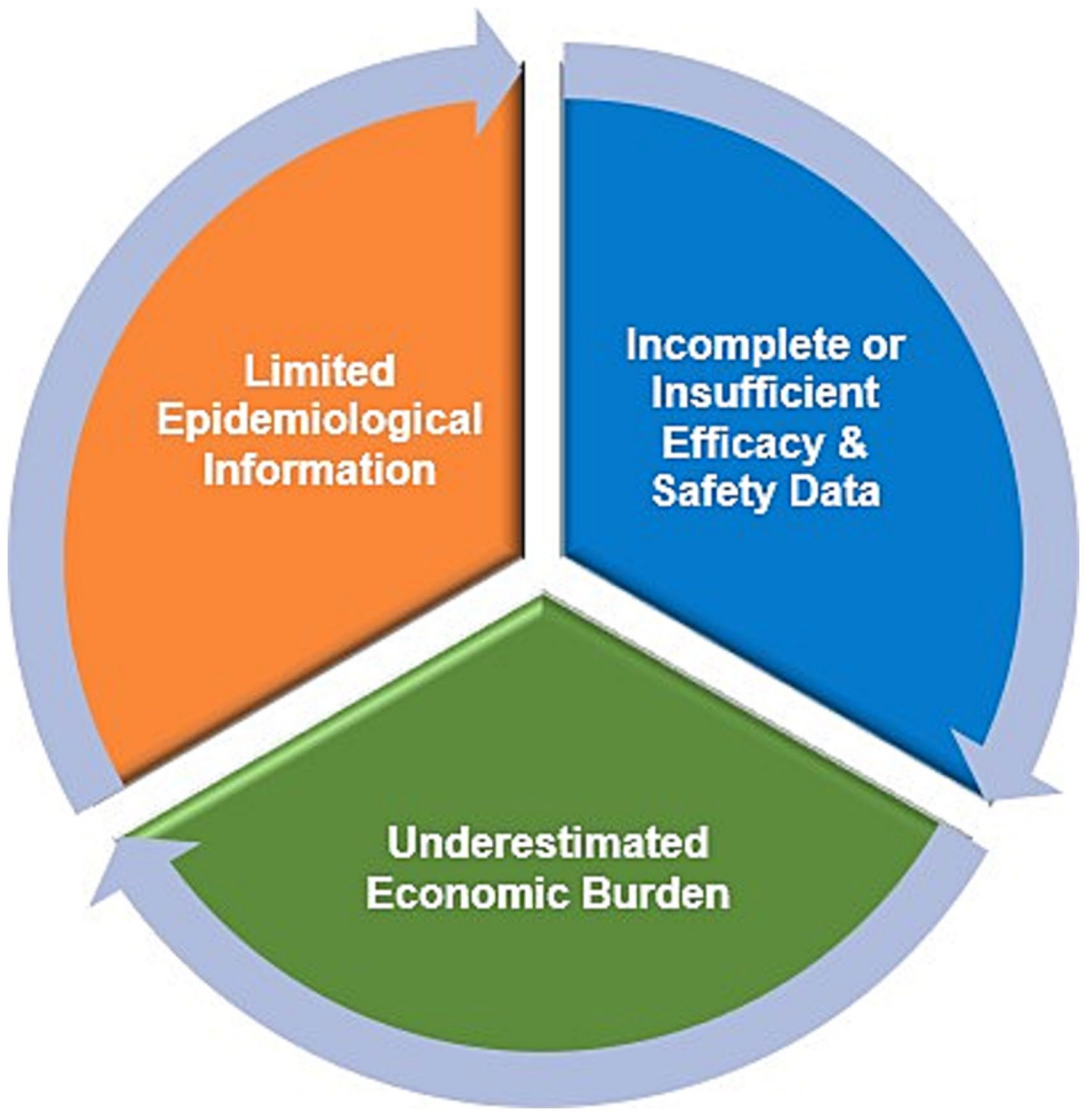
Challenges of assessing the value of orphan drugs. Data summarized from references (118, 195, 206).

**Figure 6:**
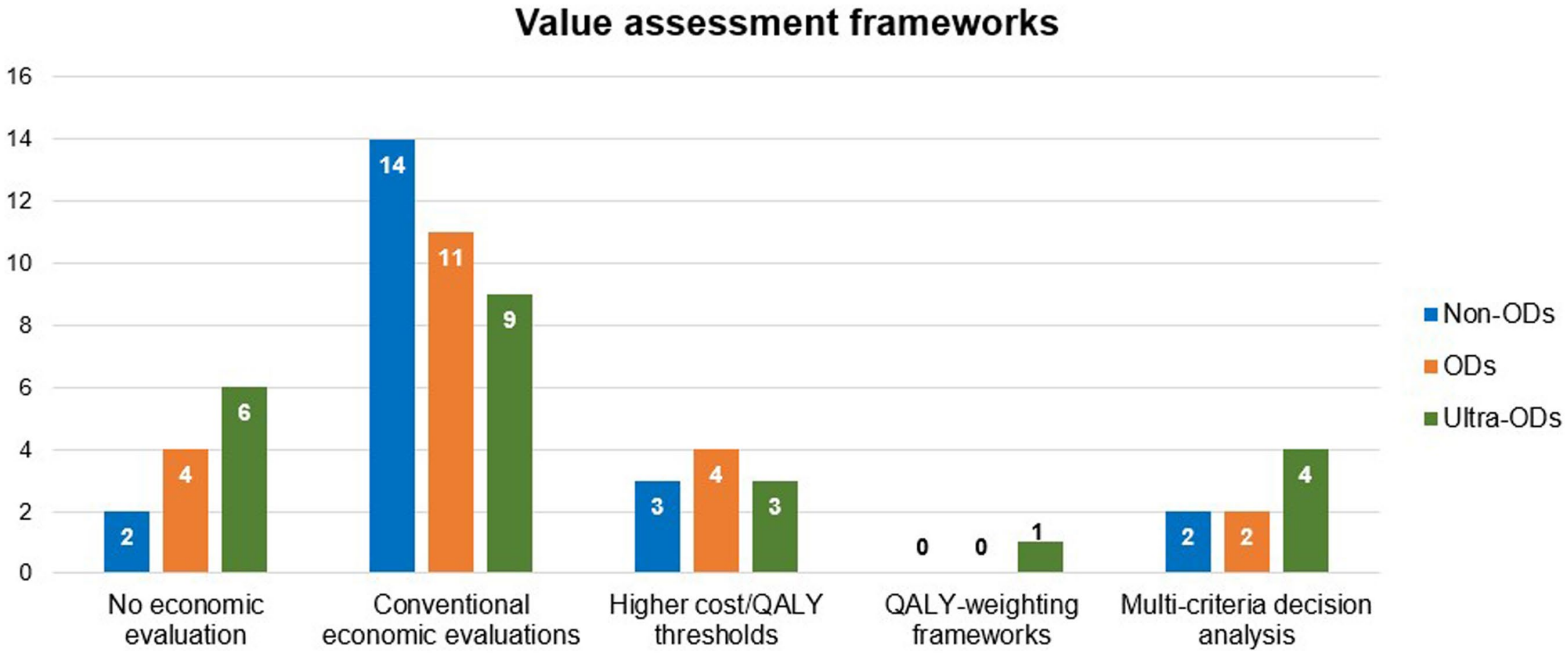
Frequency of using different types of value assessment frameworks in 18 European countries. Data summarized from reference (207). Some countries use more than 1 method for a single drug class. Non-ODs, Non-Orphan Drugs; ODs, Orphan Drugs; QALY, Quality-Adjusted Life Year, Ultra-ODs, Ultra Orphan Drugs; VAFs, Value Assessment Frameworks.

Healthcare systems in several LMICs still face challenges in making orphan drugs available and accessible to people with rare diseases due to unaffordable prices (205, 206) (Figure 5). In a review of value assessment frameworks adopted by health technology assessment (HTA) units in 18 European countries, it was found that 11 (61%) countries still evaluate orphan drugs using conventional cost-effectiveness and cost-utility analyses (207). All approaches presented in the review and their frequencies are summarized in Figure 6.

**Figure 7:**
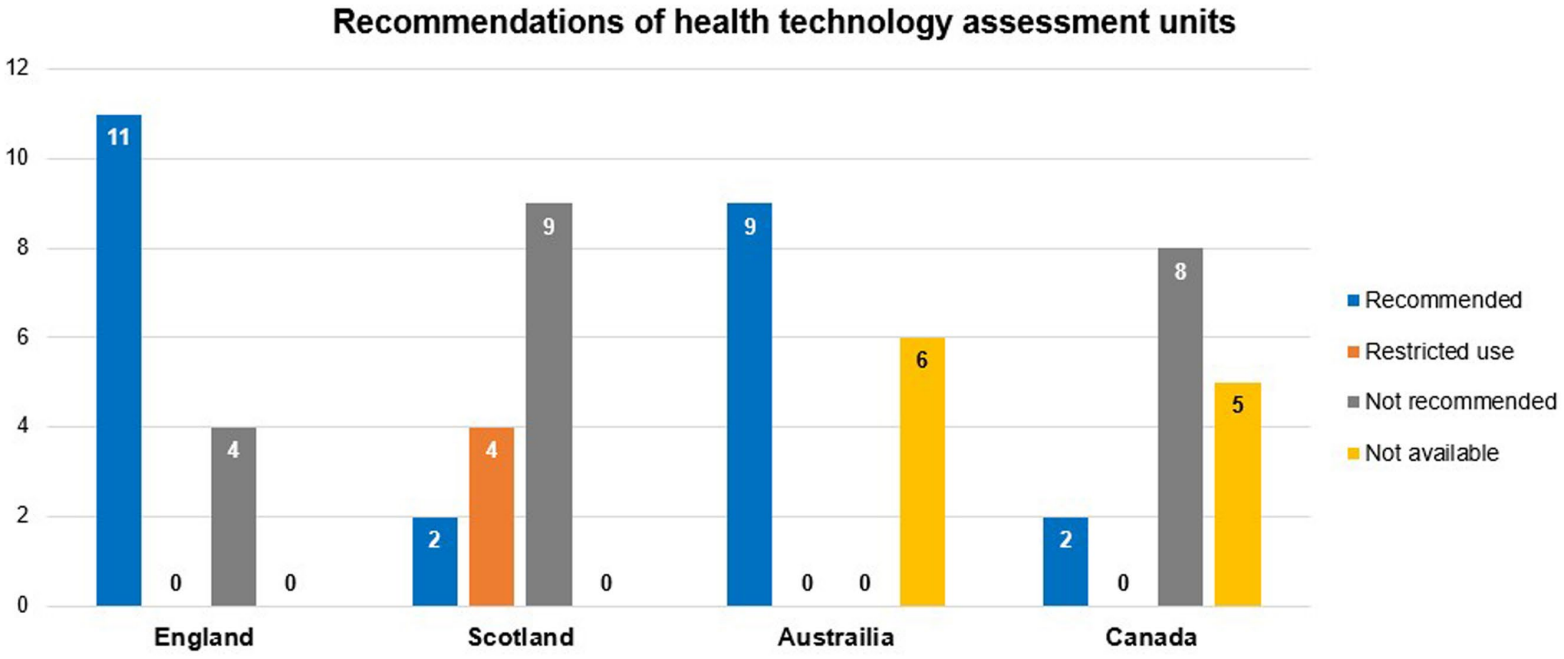
Frequency of HTA units’ recommendations for reimbursing 15 orphan drugs in four HICs from 2017 to 2018. Data summarized from reference (208). HTA, health technology assessment; HICs, high-income countries.

In a comparative analysis of the reimbursement status of 15 orphan drugs in HTA units in four high income countries (HICs) (Australia, Canada, England, and Scotland) from 2017 to 2018, significant heterogeneity in reimbursement assessment criteria and final reimbursement decisions existed among countries (Figure 7). This heterogeneity may be partly explained by variations in the prevalence data of rare diseases used in their respective countries’ assessments (208).

Moreover, a recent study confirmed a similar heterogeneity in reimbursement decisions across 12 European countries. The findings revealed that the recommendations from local HTA agencies do not significantly influence subsequent payer decisions for reimbursing orphan drugs in more than two-thirds of these countries (209). Reimbursement decisions are usually based on market access agreements, known as managed entry agreements between drug manufacturers or their agents and healthcare payers. These agreements are necessary to determine the final price of a drug when its clinical benefits are still uncertain, and its reimbursement poses a significant financial burden (210, 211). Managed entry agreements fall under two main categories, based on their purpose. If the aim is to lower the price of a new intervention or to reduce the budget impact of introducing it for the payer, it will be called a financial agreement. On the other hand, if its aim is to pay for a new intervention based on its performance, it is called an outcome- or performance-based agreement. Both categories can be further divided into two subcategories, depending on whether the agreement focuses on individual patients or a wider target population (212, 213). Each subcategory encompasses various agreement designs and templates, including special price, discount, or rebate agreements, volume- or budget-cap agreements, free initial treatment agreements, agreements for developing real-world evidence, payment-by-results agreements, conditional treatment continuation agreements, price–volume agreements, pay-back agreements, and risk-sharing agreements (210, 211, 213–216).

Equally important, the emergence of innovative funding mechanisms is crucial to ensure sustainable access to costly novel interventions (118, 211, 215–217). A report by IQVIA identified five key archetypes of such programs: (1) Blended Finance: Combines public or non-profit catalytic capital with private sector investments to promote sustainable development. This approach aligns diverse objectives—financial, social, or both—while linking funding to specific outcomes and timelines; (2) Novel Private Insurance: Offers coverage for products or services typically excluded, such as diagnostics, or for underserved patient groups, including those with pre-existing conditions; (3) Government Funding Schemes: Designed and disbursed by governments while involving contributions from multiple sectors; (4) Multi-Source Crowdfunding: Mobilizes funds from diverse stakeholders (individuals, companies, or non-profits), often incentivized through mechanisms like tax benefits; and (5) Financial Services: Provides alternative payment methods, such as credit or savings plans, facilitated by FinTech or traditional banking, enabling patients to manage costs flexibly. These innovative funding models can play a pivotal role in bridging financial gaps and improving access to advanced medical therapies (218).

As an example of rare diseases, equity in global hemophilia care remains a significant challenge. Disparities in access to laboratory and genetic diagnoses, prophylaxis and home treatment, effective treatment options, and comprehensive care still exist across different regions and countries (21–24).

### The evolving role of public health in rare diseases

#### The role of public health in the prevention of congenital rare diseases

In congenital rare diseases, primary, secondary, tertiary, and quaternary disease prevention strategies have been shown to be beneficial from public health and health economic perspectives (5–8).

In Europe, up to 15% of people living with rare diseases have rare congenital anomalies (219), which can potentially be reduced by the implementation of primary prevention strategies, such as pre-conception or pre-marital carrier genetic screening, preimplantation genetic diagnosis accompanied by *in vitro* fertilization, healthcare counseling, and educational campaigns, as well as secondary prevention strategies, such as post-conception or prenatal carrier genetic screening and newborn screening (5, 6, 220–222). Therefore, accurate and relevant epidemiological information on the prevalence, morbidity, and mortality of rare diseases is crucial in evaluating and addressing their impact on population health through public health approaches, planning and implementation of health policies, supporting the process of drug development, and the conduct of clinical trials (2, 71, 72, 223, 224).

#### Precision public health

Public health and precision medicine were initially viewed as competing fields, the former analyzes limited data from large populations with the overarching goal of improving population health, while the latter handles massive sets of data from targeted population cohorts to personalize diagnostic and therapeutic approaches based on these individuals’ needs (225). Precision public health is emerging as a bridge to reconcile these two fields, united by the common goal of achieving equitable provision of health services and reducing disparities in healthcare outcomes, especially in rare diseases (182, 226). Thus, precision public health can play an important role in utilizing big data to re-aggregate small cohorts into large-scale ones, based on biological pathway commonalities, accordingly, enabling novel personalized interventions such as pharmacogenomics, gene editing, and gene therapies to achieve effective and equitable implementation while remaining grounded in the public health values of whole population health improvement and equity (227–229).

Notably, in rare diseases, rarity varies according to demographic and geographical factors, such as rates of consanguinity for congenital disorders, and contextual factors, such as endemicity rates of contagious diseases (120). In rare genetic disorders, phenotypic variability is observed due to the varying disease severities and the different disease subtypes. This non-linear genotype–phenotype relationship is shaped by genetic and environmental disease modifiers that influence the genotype penetrance, expressivity, and pleiotropy of the causative gene of a specific genetic disorder. Understanding this relationship in rare genetic disorders using next-generation sequencing techniques, such as whole-genome sequencing, whole-exome sequencing, and targeted exome sequencing, facilitates accurate, cost-effective, and timely diagnosis (230–232). In addition, the use of novel data generation technologies, including artificial intelligence, will enhance analysis and interpretation of mass biomedical data, helping to close existing gaps in this field and advancing it into new horizons for better diagnosis and treatment of people with rare diseases (233–236).

Advances in genomic and epigenetic analyses have accelerated the research on drugs and biologics that act on disease-specific molecular pathways. Most rare diseases are monogenic disorders (182, 237, 238). Several gene-targeted therapies (GTTs) have shown great promise for rare monogenic disorders. Given the urgent needs of rare disease patients, GTTs are generating interest from the US National Institutes of Health (NIH) to hasten the drug development process for these disorders. This includes using many approaches, platforms, and master protocols to increase the logistical efficiencies for the patients to access these therapeutics (239). Moreover, FDA and the European Medicines Agency (EMA) have issued new scientific guidelines on emerging therapeutic trends including regenerative medicine therapies, gene therapies, and genetically modified cell-based therapies (240, 241).

To reinforce these efforts, the Bespoke Gene Therapy Consortium (BGTC) was recently launched as a bold partnership between the NIH, FDA, 10 pharmaceutical companies, and several non-profit organizations. It aims to optimize the development of gene therapy and fill the gaps and unmet needs of this vulnerable group (242, 243). The NIH and private partners will contribute approximately 76 million US$ over 5 years to support the projects funded by the BGTC. This includes about 39.5 million US$ from the participating NIH institutes and centers, pending the availability of funds. The National Centre for Advancing Translational Sciences (NCATS), the NIH’s lead for BGTC, is expected to contribute approximately 8 million US$ over 5 years (244).

## Discussion

The complexity of health necessitates a unique approach that acknowledges the diverse nature of health conditions and the unique needs of individuals (10). Public health plays a pivotal role in protecting and improving the health and well-being of populations worldwide (9, 37, 39, 40). Over time, the role of public health has evolved from solely focusing on the prevention of infectious diseases to reducing the burden of non-communicable diseases and recognizing the needs of individuals with rare diseases (6, 220–222). Notably, the global landscape of rare diseases presents significant challenges, as there is currently no unified global definition for the prevalence threshold of a rare disease (63–73). With over 10,000 identified rare diseases (120, 245) impacting approximately 450 million people globally (71), it is essential to gather accurate epidemiological information to understand and address their impact on population health effectively.

In this review, we selected hemophilia as our case study because it is one of the most costly rare diseases to manage over the patient’s lifetime (145–148). Moreover, hemophilia is diagnosed shortly after birth and its health outcomes rely heavily on treatment accessibility (124, 125). Without appropriate treatment, people with severe hemophilia will develop long-term and debilitating musculoskeletal complications due to frequent bleeding and their life expectancy will be severely compromised, with early mortality during childhood and adolescence, which is the case in many LMICs (16, 17). In HICs, gene therapy is a viable one-time treatment option for hemophilia, with several vectors approved by the FDA and other regulatory bodies worldwide (18, 20). However, health systems around the globe are still struggling with the pricing, funding, and reimbursement frameworks of gene therapies especially given the expensive upfront payments (19). For these reasons—among others, public health can potentially enhance the access of people with hemophilia to the available treatment options worldwide, through surveillance, advocacy, and planning (246–248).

Public health plays a vital role in managing rare diseases on multiple levels, beginning with the prevention of genetically linked congenital disorders and addressing health inequities that disproportionately impact patients with rare diseases (71, 249). Evidence from HICs has shown that approximately 70% of congenital disorders are preventable or treatable when the appropriate public health measures are implemented (250, 251). Although many congenital diseases that present at birth have a genetic nature (e.g., osteogenesis imperfecta), others develop due to a variety of factors such as environmental risk factors, problems during development, or birth itself (e.g., fetal alcohol syndrome). Thus, congenital and genetic diseases are not identical (251, 252). Yet, the success of public health in controlling congenital disorders with genetic nature should motivate the global community to implement disease prevention strategies to reduce disease prevalence and burden (193). Additionally, to achieve better population health, it is essential to address the determinants of health and health inequities, including the genetic determinants that affect individuals with rare diseases (253, 254).

At the policy making level, legislative and regulatory support has facilitated the development and approval of diagnostic and therapeutic agents for rare diseases, leading to significant advancements in the treatment landscape (255). For example, the cost of developing new treatments for rare diseases may be lowered by approximately 60% when implementing policies and regulations for accelerated drug approval, which also shortens the time to approval to one-third (256). Incentives to drug manufacturers have attracted remarkable investments in developing numerous orphan therapeutic products in recent years (203). Therefore, it is crucial that governmental health directorates realize that investment in research, development, and regulatory reforms for better care for people with rare diseases is highly lucrative from economic and clinical perspectives (257). This process should be ongoing to ensure the sustainability of innovation in the field of rare diseases (258), and aspire to shift the focus of developing orphan drugs from reaching a profitability threshold by marketing those drugs in specific markets, especially HICs, to achieving an equitable environment through attaining comparable health outcomes across various diseases and different income levels (259).

Despite the remarkable progress that has occurred in the legislative and regulatory domains in the current century, greater investment and innovation in drug discovery and market access pathways in LMICs and HICs are still needed (183, 202, 260–262). Patient access to these expensive medications, even in HICs, is not guaranteed unless alternative value-based assessment approaches with complementary elements, as well as innovative pricing and reimbursement schemes, are proposed by relevant HTA units (118, 211, 217). Additionally, payers should adhere to HTA agencies recommendations in line with evidence-based decision-making to facilitate timely patient access to newly discovered orphan drugs (215, 216). By implementing such measures, the principles of health equity can be upheld, ensuring that individuals with rare diseases are fully included as valued members of society (3, 263).

Overall, a key factor to the success of public health policies is developing feasible implementation strategy with a clear and ongoing monitoring and evaluation plan to ensure that these policies are translated into regulatory measures through value assessment frameworks, and support healthcare systems and healthcare professionals while implementing them (65, 81, 176, 185, 211, 258, 264–266). Yet, many countries, especially LMICs face several challenges in enacting legislation, developing regulations, and implementing policies to support the diagnosis and treatment of people with rare diseases (63, 65, 66, 81, 82, 188, 264, 267, 268). These challenges include a lack of awareness about the burden of rare diseases, insufficient financial and human resources, inadequate health systems and infrastructure, absence of national policies and strategies for managing rare diseases and lack of a feasible implementation plan to translate policies into actions. All these limitations compromise patient access to diagnostic and therapeutic tools, which leads to increased morbidity and mortality (68, 81, 182, 226, 261, 269, 270). Efforts are underway to address these challenges through international collaborations, capacity-building initiatives, and raising awareness about the impact of rare diseases on public health (271–273).

**Figure 8:**
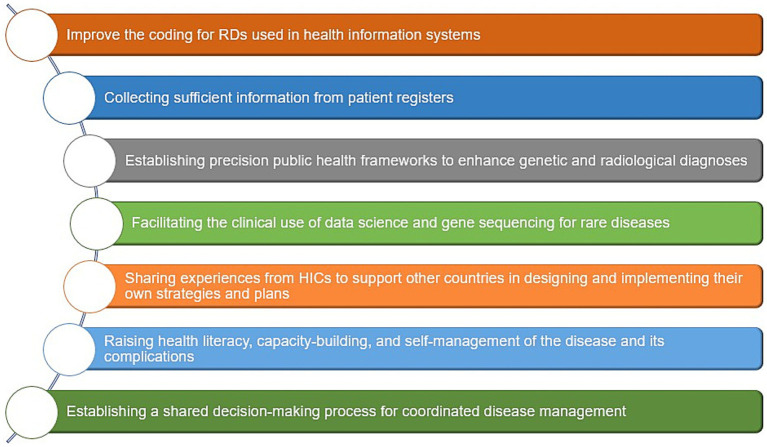
Suggested public health approaches to overcome challenges of RDs in LMICs. Data summarized from references (1, 68, 73, 80, 81, 98, 104, 176, 182, 226, 230, 232, 237, 261, 269, 270, 274–278). HICs, High-income countries; LMICs, low- and middle-income countries; RD(s), rare disease(s).

Suggested approaches to overcome challenges in LMICs include (1) improving coding for rare diseases in patients’ medical records used in health information systems (68, 182, 274), (2) collecting sufficient information through patient registers (1, 68, 98, 176), (3) establishing precision public health frameworks to enhance genetic and radiological diagnoses (182, 226, 237), (4) facilitating the clinical use of data science and gene sequencing for rare diseases, which improves the quality of epidemiological data and informs public health policy (80, 182, 230, 232), (5) sharing experiences from HICs that have developed and implemented efficient policies and strategies, to support other countries in designing their own (73, 81, 104, 261, 269, 270), (6) raising health literacy, capacity-building, and self-management of the disease and its complications (275, 276), and finally (7) establishing a shared decision-making process for coordinated disease management (68, 277, 278) (Figure 8).

In the context of hemophilia, public health can play a crucial role in improving hemophilia care by addressing the gaps in the availability and accuracy of epidemiological data, advocating for equitable access to treatment for better hemophilia care (21–24), and implementing evidence-based interventions into routine clinical practice (164, 247, 248). Public health approaches—including health promotion; primordial, primary, secondary, tertiary, and quaternary disease prevention strategies (6, 279); public health surveillance; and policy development and implementation—can improve the overall management and outcomes of hemophilia care (162, 164, 280). Furthermore, public health efforts should focus on overcoming barriers such as inadequate healthcare infrastructure, shortages of trained healthcare professionals, and affordability challenges. Collaborative initiatives involving governments, healthcare systems, professional and patient organizations, and international stakeholders are essential to ensuring equitable access to comprehensive healthcare services for all individuals with hemophilia, regardless of geographic location or socioeconomic status (21–24). As such, by integrating public health principles into hemophilia care, the focus can shift toward more comprehensive, patient-centered, cost-effective approaches that address the broader health needs of individuals with hemophilia and their families to improve their health-related quality of life and achieve sustainable physical and mental health (246, 281).

Thus, to achieve optimal hemophilia care, several priorities need to be met. First, provide laboratory, radiological, and genetic diagnosis, including carrier detection and newborn screening. Second, treat acute bleeds, including serious and life-threatening bleeds. Third, prevent musculoskeletal complications by offering prophylactic treatment to people with hemophilia. Fourth, prevent blood-borne infections by providing safe coagulation factors and other hemostatic therapies. Fifth, delay, reduce, or prevent inhibitor development to avoid putting an additional disease burden on people with hemophilia. Sixth, restore musculoskeletal health by providing the appropriate physiotherapy services and performing the required surgeries. Seventh, offer psychosocial support to patients and families to reduce the humanistic disease burden and improve health-related quality of life (139, 282, 283). And last, create pathways for access to novel therapies through innovative pricing and reimbursement schemes (284, 285).

Brazil stands out as a notable success story among LMICs, demonstrating how targeted policy efforts can improve care for rare diseases (286, 287). Brazil, ranks fourth in the number of people diagnosed with hemophilia worldwide, following China, India, and the USA (13). The Brazilian national health system offers full reimbursement of medical care for people with rare diseases following the establishment of a national policy and treatment guidelines for comprehensive care (81, 288, 289). This policy formulation was complemented by the enactment of legislation and the development of regulations to support the implementation and enforcement of these health policies. Despite this legislative and regulatory support, funding and patient access to treatment remains subject to the availability of appropriate funding (73, 290).

In rare diseases, the availability of clinical practice guidelines facilitates the diagnosis and treatment of these rare conditions, as well as the implementation of preventive public health measures (291, 292). The latest hemophilia management guidelines issued by the World Federation of Hemophilia acknowledge low-dose prophylaxis as a superior treatment option over episodic treatment for people with hemophilia living in low-resource countries with limited access to clotting factor replacement therapies (139).

To achieve equitable and sustainable physical and mental health outcomes for individuals with rare diseases, including hemophilia, it is imperative to adopt innovative, transparent, and evidence-, outcome-, and value-based pricing, reimbursement, and funding strategies for orphan medicinal products to lower the healthcare economic burden and out-of-pocket expenditure on healthcare (183, 210, 214, 216, 293–295). Because the proportion of non-healthcare expenditure on the management of rare diseases is significantly high, it is crucial to use a societal perspective when estimating the economic burden of rare diseases on patients and caregivers (117–119, 296).

**Figure 9:**
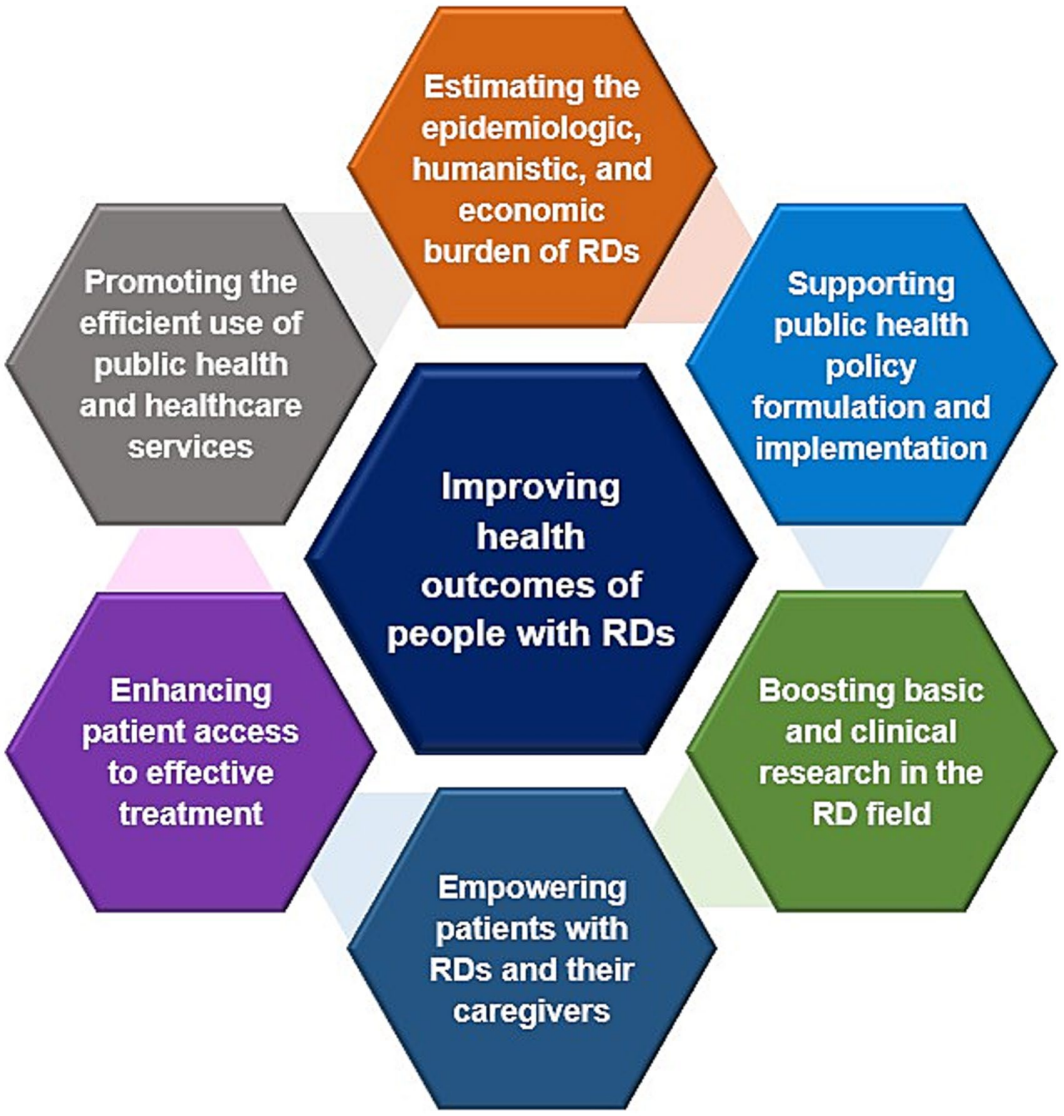
Objectives of public health in supporting the RDs community. Data summarized from references (3, 64, 68, 72, 115, 116, 119, 140, 142, 188, 202, 211, 248, 249, 255–257, 265, 269, 284, 285, 301–303). RD(s), rare disease(s).

The objectives of public health in supporting people with rare diseases should focus on (1) accurately estimating the epidemiological, and economic burden of rare diseases through expanding newborn screening programs, benefiting from genetic diagnostics, strengthening surveillance systems, and assessing costs and cost-effectiveness from a comprehensive societal perspective (115, 116, 140, 142, 249), (2) supporting public health policy formulation and implementation through integrative actions inside and outside health systems (68, 72), (3) boosting basic and clinical research in the rare disease field through international collaborations to accelerate clinical trials and by establishing specialized clinics and centers of excellence (245, 297–300), (4) empowering patients with rare diseases and their caregivers through implementing a comprehensive psychosocial support plan comprising counseling programs, caregiver support networks, and mental health services (64, 202, 248), (5) enhancing patient access to effective treatment through novel pricing and reimbursement schemes (188, 211, 255–257, 269, 284, 285, 301), (6) promoting the efficient use of public health and healthcare services through strengthening health systems and optimizing allocation of resources (3, 302), and finally (7) improving health outcomes of people with rare diseases (119, 265, 303) (Figure 9).

The latter and ultimate objective can be strengthened by the invention and availability of innovative therapies, including cell and gene therapies (301, 304–306). In hemophilia, the treatment landscape has dramatically evolved over the past two decades with the licensure of several emerging treatment options, such as extended half-life recombinant factor VIII and FIX products, non-factor replacement subcutaneous agents, and gene therapies (307). These advanced and innovative treatment options have also raised the bar for more ambitious treatment outcomes, making people with hemophilia realize a normal and bleed-free life (308). Despite this scientific and clinical progress in hemophilia care, patient access to these evolving treatment options is still limited to HICs with strong public health systems, sufficient economic resources, and efficient disease awareness and advocacy (22). LMICs with lower capabilities should identify the minimal requirements to provide the best possible care for their people with hemophilia (17). Therefore, a structured plan should be designed, with specific roles and responsibilities for each stakeholder to achieve quality and sustainable care for people with hemophilia (309). Effective collaborative efforts between all concerned stakeholders and strong public health support at best across borders and between LMICs and HICs, can facilitate and overcome these challenges (245, 299, 310, 311).

## Conclusion

Public health has evolved to play a vital role in protecting and improving the health and well-being of people globally. Initially focused on preventing infectious diseases, the scope of public health has expanded to address non-communicable diseases and the unique needs of individuals with rare diseases. Addressing the genetic determinants of health and health inequities is essential to providing better care for those with rare diseases. The global landscape of rare diseases presents significant challenges, as there is no universal definition of rarity based on disease prevalence. However, legislative and regulatory support in HICs has facilitated the development and approval of diagnostics and treatments for several rare diseases leading to important advancements. In contrast, many LMICs face obstacles in enacting legislation, developing regulations, and implementing policies to support rare disease diagnosis and treatment. More investment and innovation in drug discovery and market access pathways are still needed in both LMICs and HICs. Ensuring the translation of public health policies into regulatory measures, and in turn implementing and regularly evaluating these measures to assess their effectiveness is important to facilitate the provision of high-quality care for vulnerable populations with rare diseases. Clinical practice guidelines also facilitate diagnosis, treatment, and preventive public health interventions. In the case of hemophilia, public health can play a pivotal role. This includes addressing gaps in epidemiological data, advocating for equitable access to treatment, and implementing evidence-based interventions into routine clinical practice to improve hemophilia care. Overall, public health has a crucial role in ensuring that individuals with rare diseases receive the care and support they need through a multifaceted approach addressing genetic factors, health inequities, legislative frameworks, and evidence-based practices.
