# Supplementary material for: The role of public health in rare diseases: hemophilia as an example
Source: Front Public Health. 2025 Mar 20;13:1450625. doi: 10.3389/fpubh.2025.1450625 (PMC11965367; doi:10.3389/fpubh.2025.1450625)
Supplement: Supplementary file 1 [file Data_Sheet_1.pdf]

frontiers

The role of public health in rare diseases: Hemophilia as an example

Supplementary Material

Table of content

1. Supplementary Table 1 --- 2
2. Supplementary Table 2 -- 36
3. References --- 40

1 **Supplementary Table 1.** Sources of evidence identified for this review, n = 315.

Ser.	Reference & year	Reference title	Reference type	Publication type	Evidence type	Evidence category
**1**	Abuhadida et al, 2022	Return on Investment from the Prevention of Orphan Diseases in Kuwait	Topic-related	Journal article	Original research article	Primary
**2**	Adachi et al, 2023	Enhancing Equitable Access to Rare Disease Diagnosis and Treatment around the World: A Review of Evidence, Policies, and Challenges	Topic-related	Journal article	Literature review	Secondary
**3**	Adama et al, 2023	The psychosocial impact of rare diseases among children and adolescents attending mainstream schools in Western Australia	Topic-related	Journal article	Original research article	Primary
**4**	Adams et al, 2024	Addressing diagnostic gaps and priorities of the global rare diseases community: Recommendations from the IRDiRC diagnostics scientific committee	Topic-related	Journal article	Recommendations	Secondary
**5**	Ali et al, 2021	The quality evaluation of rare disease registries—an assessment of the essential features of a disease registry	Topic-related	Journal article	Original research article	Primary
**6**	American Public Health Association, 2023	What is public health?	Topic-related	Professional website	Online briefing	Secondary
**7**	American thrombosis and hemostasis	Community Counts: CDC Public Health Surveillance Project For Bleeding Disorders	Topic-related	Professional website	Online briefing	Secondary

	network, 2023					
**8**	Andreu et al, 2022	The burden of rare diseases: an economic evaluation	Topic-related	Report	Overview	Secondary
**9**	Angelis et al, 2015	Socio-economic burden of rare diseases: A systematic review of cost of illness evidence	Topic-related	Journal article	Systematic review	Secondary
**10**	Angin et al, 2024	Coding undiagnosed rare disease patients in health information systems: recommendations from the RD-CODE project	Topic-related	Journal article	Recommendations	Secondary
**11**	Annemans et al, 2017	Recommendations from the European Working Group for Value Assessment and Funding Processes in Rare Diseases (ORPH-VAL)	Topic-related	Journal article	Recommendations	Secondary
**12**	Armeni et al, 2021	Reflections on the Importance of Cost of Illness Analysis in Rare Diseases: A Proposal	Topic-related	Journal article	Literature review	Secondary
**13**	Arnold et al, 2015	The role of globalization in drug development and access to orphan drugs: orphan drug legislation in the US/EU and in Latin America	Topic-related	Journal article	Literature review	Secondary
**14**	Atkins and Padgett, 2024	Living with a Rare Disease: Psychosocial Impacts for Parents and Family Members – a Systematic Review	Topic-related	Journal article	Literature review	Secondary
**15**	Atrash and Carpentier, 2012	The evolving role of public health in the delivery of health care	Topic-related	Journal article	Overview	Secondary

**16**	Austin et al, 2018	Future of Rare Diseases Research 2017-2027: An IRDiRC Perspective.	Topic-related	Journal article	Literature review	Secondary
**17**	Australian Government, 2015	Orphan drugs program discussion paper	Topic-related	Governmental document	Governmental report	Secondary
**18**	Aymé et al, 2008	Empowerment of patients: lessons from the rare diseases community	Topic-related	Journal article	Perspective	Secondary
**19**	Baba et al, 2018	Public Health, Population Health, and Planning: Ideas to Improve Communities	Topic-related	Journal article	Perspective	Secondary
**20**	Bajaj et al, 2019	Genomics of rare genetic diseases—experiences from India	Topic-related	Journal article	Literature review	Secondary
**21**	van Balen et al, 2021	Patient-relevant health outcomes for hemophilia care: Development of an international standard outcomes set	Topic-related	Journal article	Systematic review	Secondary
**22**	Barton and Grant, 2006	A health map for the local human habitat	Topic-related	Journal article	Perspective	Secondary
**23**	Baynam et al, 2017	Improved diagnosis and care for rare diseases through implementation of precision public health framework	Topic-related	Textbook	Book chapter	Secondary
**24**	Baynam et al, 2020	A call for global action for rare diseases in Africa	Topic-related	Journal article	Commentary	Secondary
**25**	Belzer et al, 2022	Psychosocial Considerations for the Child with Rare Disease: A Review with Recommendations and Calls to Action	Topic-related	Journal article	Literature review	Secondary

**26**	Berntorp et al, 2021	Haemophilia	Topic-related	Journal article	Literature review	Secondary
**27**	Bhattacharya et al, 2021	Rare diseases research and policy in Australia: On the journey to equitable care	Topic-related	Journal article	Perspective	Secondary
**28**	Bilkey et al, 2019	Optimizing Precision Medicine for Public Health	Topic-related	Journal article	Literature review	Secondary
**29**	Birkelund, 2019	Rare disease thresholds-An analysis of different definitions, laws and arguments	Topic-related	Thesis	Literature review	Secondary
**30**	Blonda et al, 2021	How to Value Orphan Drugs? A Review of European Value Assessment Frameworks	Topic-related	Journal article	Literature review	Secondary
**31**	Bolous et al, 2022	Gene Therapy and Hemophilia: Where Do We Go from Here?	Topic-related	Journal article	Literature review	Secondary
**32**	Bouwman et al, 2020	Regulatory issues for orphan medicines: A review	Topic-related	Journal article	Literature review	Secondary
**33**	Bradley et al, 2018	Toward a Contemporary Definition of Health	Topic-related	Journal article	Perspective	Secondary
**34**	Braveman and Gottlieb, 2014	The social determinants of health: it's time to consider the causes of the causes	Topic-related	Journal article	Literature review	Secondary
**35**	Brooks, 2024	The Bespoke Gene Therapy Consortium: facilitating development of AAV gene therapies for rare diseases	Topic-related	Journal article	Commentary	Secondary
**36**	Bruckner-Tuderman, 2021	Epidemiology of rare diseases is important	Topic-related	Journal article	Commentary	Secondary

**37**	Budhwar et al, 2016	Regulations of orphan drugs in USA, EU and India-A comparative study	Topic-related	Journal article	Literature review	Secondary
**38**	Burke et al, 2021a	Clinical, humanistic, and economic burden of severe hemophilia B in the United States: Results from the CHESS US and CHESS US+ population surveys	Topic-related	Journal article	Original research article	Primary
**39**	Burke et al, 2021b	Clinical, humanistic, and economic burden of severe haemophilia B in adults receiving factor IX prophylaxis: findings from the CHESS II real-world burden of illness study in Europe	Topic-related	Journal article	Original research article	Primary
**40**	Byams et al, 2023	Building the foundation for a community-generated national research blueprint for inherited bleeding disorders: research priorities in health services; diversity, equity, and inclusion; and implementation science	Topic-related	Journal article	Perspective	Secondary
**41**	CADTH, 2016	Drugs for rare diseases: evolving trends in regulatory and health technology assessment perspectives	Topic-related	Governmental document	Governmental report	Secondary
**42**	Capili, 2021	Cross-Sectional Studies	Topic-related	Journal article	Overview	Secondary
**43**	Capital Area Public Health Network, 2023	What is public health?	Topic-related	Professional website	Online briefing	Secondary

**44**	Carvalho et al, 2021	Patient access to gene therapy medicinal products: a comprehensive review	Topic-related	Journal article	Systematic review	Secondary
**45**	CDC, 2015	Definition of Policy	Topic-related	Official website	Online briefing	Secondary
**46**	CDC, 2018	Introduction to Public Health Surveillance	Topic-related	Official website	Online briefing	Secondary
**47**	CDC, 2020	The 10 Essential Public Health Services	Topic-related	Official website	Online briefing	Secondary
**48**	CDC, 2022	Social Determinants of Health at CDC	Topic-related	Official website	Online briefing	Secondary
**49**	CDC, 2023	What is Public Health?	Topic-related	Official website	Online briefing	Secondary
**50**	Chan et al, 2020	Access and Unmet Needs of Orphan Drugs in 194 Countries and 6 Areas: A Global Policy Review With Content Analysis	Topic-related	Journal article	Systematic review	Secondary
**51**	Chang et al, 2022	A survey of registered pharmacological clinical trials on rare neurological diseases in children in 2010-2020	Topic-related	Journal article	Literature review	Secondary
**52**	Chigbu et al, 2023	The Science of Literature Reviews: Searching, Identifying, Selecting, and Synthesising	Methodological	Journal article	Overview	Secondary
**53**	Chernyi et al, 2024	Recent Advances in Gene Therapy for Hemophilia: Projecting the Perspectives	Topic-related	Journal article	Literature review	Secondary
**54**	Choi, 2012	The past, present, and future of public health surveillance	Topic-related	Journal article	Literature review	Secondary

**55**	Choon et al, 2024	Artificial intelligence and database for NGS-based diagnosis in rare disease	Topic-related	Journal article	Literature review	Secondary
**56**	Chung et al, 2022	Rare disease emerging as a global public health priority	Topic-related	Journal article	Literature review	Secondary
**57**	Chung et al, 2023	Socio-economic costs of rare diseases and the risk of financial hardship: a cross-sectional study	Topic-related	Journal article	Original research article	Primary
**58**	Coffin et al, 2023	The World Federation of Hemophilia World Bleeding Disorders Registry: insights from the first 10,000 patients	Topic-related	Journal article	Original research article	Primary
**59**	Columbia University, 2021	Public Health Policy: Definition, Examples, and More	Topic-related	Official website	Online briefing	Secondary
**60**	Committee for Advanced Therapies, 2021	Guideline on quality, non-clinical and clinical aspects of medicinal products containing genetically modified cells - Revision 1	Topic-related	Official website	Online briefing	Secondary
**61**	Congressional Budget Office, 2023	The Demographic Outlook: 2023 to 2053	Topic-related	Official website	Online briefing	Secondary
**62**	Coppola et al, 2022	Current choices and management of treatment in persons with severe hemophilia A without inhibitors: a mini-Delphi consensus	Topic-related	Journal article	Recommendations	Secondary
**63**	Cornel et al, 2021	Neonatal and carrier screening for rare diseases: how innovation challenges screening criteria worldwide	Topic-related	Journal article	Overview	Secondary

**64**	countryeconomy.com, n.d.	Japan - Population	Topic-related	Professional website	Online briefing	Secondary
**65**	County of Los Angeles, 2004	Public Health Core Functions and Essential Services	Topic-related	Governmental document	Online briefing	Primary
**66**	Cox et al, 2022	Editorial: Prevention, diagnosis and treatment of rare disorders	Topic-related	Journal article	Editorial	Secondary
**67**	Cunico et al, 2023	Initiatives to promote access to medicines after publication of the Brazilian Policy on the Comprehensive Care of People with Rare Diseases	Topic-related	Journal article	Literature review	Secondary
**68**	Czech et al, 2019	A Review of Rare Disease Policies and Orphan Drug Reimbursement Systems in 12 Eurasian Countries	Topic-related	Journal article	Systematic review	Secondary
**69**	Czech et al, 2019	Bridging East with West of Europe – A comparison of orphan drug policies in Poland, Russia and the Netherlands	Topic-related	Journal article	Systematic review	Secondary
**70**	Czerska and Skweres-Kuchta, 2021	Integrative medicine as a new treatment model and the future of health care systems in the world in the context of rare diseases	Topic-related	Journal article	Overview	Secondary
**71**	Dale et al, 2014	Rare Disease Registries	Topic-related	Textbook	Book chapter	Secondary
**72**	Dang, 2023	Real-World Evidence: A Primer	Topic-related	Journal article	Literature review	Secondary
**73**	David et al, 2023	The Plight of Rare Diseases in Southern Africa: Health	Topic-related	Journal article	Perspective	Secondary

		and Social Services Policy Recommendations				
**74**	Decker et al, 2023	Novel approach to decision making for orphan drugs	Topic-related	Journal article	Overview	Secondary
**75**	Delaye et al, 2022	Valuing the "Burden" and Impact of Rare Diseases: A Scoping Review	Topic-related	Journal article	Scoping review	Secondary
**76**	Derayeh et al, 2018	National information system for rare diseases with an approach to data architecture: A systematic review	Topic-related	Journal article	Systematic review	Secondary
**77**	Devleesschauwer et al, 2014	Calculating disability-adjusted life years to quantify burden of disease	Topic-related	Journal article	Overview	Secondary
**78**	Dharmarajan et al, 2014	Out-of-pocket and catastrophic expenditure on treatment of haemophilia by Indian families	Topic-related	Journal article	Original research article	Primary
**79**	Dharssi et al, 2017	Review of 11 national policies for rare diseases in the context of key patient needs	Topic-related	Journal article	Literature review	Secondary
**80**	Dias et al, 2023	Rarecare: A policy perspective on the burden of rare diseases on caregivers in Latin America	Topic-related	Journal article	Literature review	Secondary
**81**	Doherty and Kelley, 2023	Bleeding Disorders	Topic-related	Textbook	Book chapter	Secondary
**82**	Dolan et al, 2014	Enhancing haemophilia care through registries	Topic-related	Journal article	Literature review	Secondary
**83**	Dolley, 2018	Big Data’s Role in Precision Public Health	Topic-related	Journal article	Literature review	Secondary

**84**	Dugger et al, 2018	Drug development in the era of precision medicine	Topic-related	Journal article	Literature review	Secondary
**85**	Edelstein and Yoder, 2016	The Child in Context of the Family, Community, and Society	Topic-related	Textbook	Book chapter	Secondary
**86**	Edu et al, 2024	Burden of rare diseases in Africa: Recommendations for improving access to medications and healthcare	Topic-related	Journal article	Letter to the Editor	Secondary
**87**	Eichler et al, 2023	Orphan drugs' clinical uncertainty and prices: Addressing allocative and technical inefficiencies in orphan drug reimbursement	Topic-related	Journal article	Overview	Secondary
**88**	El-Sayed and Bolous, 2024	Economic Burden of Haemophilia from a Societal Perspective: A Scoping Review	Topic-related	Journal article	Scoping review	Secondary
**89**	Encina et al, 2019	Rare diseases in Chile: challenges and recommendations in universal health coverage context	Topic-related	Journal article	Position statement	Secondary
**90**	Epps et al, 2022	Global Regulatory and Public Health Initiatives to Advance Pediatric Drug Development for Rare Diseases	Topic-related	Journal article	Literature review	Secondary
**91**	Ertok et al, 2023	RARE DISEASES IN TURKEY	Topic-related	Report	Overview	Secondary
**92**	EUROCAT Central Registry, 2012	EUROCAT Special Report: Congenital Anomalies are a Major Group of Mainly Rare Diseases	Topic-related	Official document	Official report	Secondary
**93**	European Commission, 2018	INNOVATIVE PAYMENT MODELS FOR HIGH-COST INNOVATIVE	Topic-related	Official document	Official report	Secondary

		MEDICINES. Report of the Expert Panel on effective ways of investing in Health (EXPH)				
**94**	European Commission, 2022	Draft proposal for a European Partnership under Horizon Europe Rare Diseases	Topic-related	Official document	Official proposal	Secondary
**95**	European Commission, 2023	Orphan medicinal products	Topic-related	Official website	Online briefing	Secondary
**96**	European Economic and Social Committee, 2023	Opinion of the European Economic and Social Committee on Ensuring strong European solidarity for rare disease patients	Topic-related	Official document	Official report	Secondary
**97**	European Parliament, 2023	Public health - Fact Sheets on the European Union	Topic-related	Official website	Online briefing	Secondary
**98**	European Union, 2000	Regulation (EC) No 141/2000 of the European Parliament and of the Council of 16 December 1999 on orphan medicinal products	Topic-related	Official document	Regulation	Primary
**99**	Eurostat, 2006	Population statistics	Topic-related	Official document	Official report	Primary
**100**	Eurostat, 2023	EU population increases again after two years decrease	Topic-related	Official website	Online briefing	Primary
**101**	Fantini and Vaccaro, 2019	Value based healthcare for rare diseases: efficiency, efficacy, equity	Topic-related	Journal article	Literature review	Secondary
**102**	Fermaglich and Miller, 2023	A comprehensive study of the rare diseases and conditions targeted by orphan drug designations and approvals over the	Topic-related	Journal article	Original research article	Primary

		forty years of the Orphan Drug Act				
**103**	Ferrelli et al, 2017	Sustainable public health systems for rare diseases	Topic-related	Journal article	Literature review	Secondary
**104**	Fidan et al, 2023	Carrier screening programs for rare diseases in developed countries and the case of Turkey: A systematic review	Topic-related	Journal article	Systematic review	Secondary
**105**	Fischer et al, 2014	Prospective observational cohort studies for studying rare diseases: the European PedNet Haemophilia Registry	Topic-related	Journal article	Original research article	Primary
**106**	Fornari et al, 2024	The psychosocial impact of haemophilia from patients' and caregivers' point of view: The results of an Italian survey	Topic-related	Journal article	Original research article	Primary
**107**	Fox and Booth, 2024	Improving access to gene therapy for rare diseases	Topic-related	Journal article	Perspective	Secondary
**108**	Franco, 2013	Orphan drugs: the regulatory environment	Topic-related	Journal article	Literature review	Secondary
**109**	Gahl et al, 2021	Essential list of medicinal products for rare diseases: recommendations from the IRDiRC Rare Disease Treatment Access Working Group	Topic-related	Journal article	Perspective	Secondary
**110**	Gammie et al, 2015	Access to Orphan Drugs: A Comprehensive Review of Legislations, Regulations and Policies in 35 Countries	Topic-related	Journal article	Systematic review	Secondary
**111**	García-Pérez et al, 2021	Cost-of-illness studies in rare diseases: a scoping review	Topic-related	Journal article	Scoping review	Secondary

**112**	Gittus et al, 2023	Barriers and facilitators to the implementation of guidelines in rare diseases: a systematic review	Topic-related	Journal article	Systematic review	Secondary
**113**	Giugliani et al, 2024	Rare disease landscape in Brazil: report of a successful experience in inborn errors of metabolism	Topic-related	Journal article	Literature review	Secondary
**114**	Gogia et al, 2023	New directions to develop therapies for people with hemophilia	Topic-related	Journal article	Literature review	Secondary
**115**	Gómez et al, 2021	Addressing health equity and social determinants of health through healthy people 2030	Topic-related	Journal article	Perspective	Secondary
**116**	Graham, 2004	Social determinants and their unequal distribution: clarifying policy understandings	Topic-related	Journal article	Perspective	Secondary
**117**	Griffiths et al, 2005	Public health in practice: the three domains of public health	Topic-related	Journal article	Perspective	Secondary
**118**	Groseclose and Buckeridge, 2017	Public Health Surveillance Systems: Recent Advances in Their Use and Evaluation	Topic-related	Journal article	Literature review	Secondary
**119**	Haendel et al, 2020	How many rare diseases are there?	Topic-related	Journal article	Commentary	Secondary
**120**	Hall and Carlson, 2014	The current status of orphan drug development in Europe and the US	Topic-related	Journal article	Literature review	Secondary
**121**	Halley et al, 2022	A call for an integrated approach to improve efficiency, equity and sustainability in rare disease research in the United States	Topic-related	Journal article	Commentary	Secondary

**122**	Hamilton and Hopkins, 2019	Using technologies for data collection and management	Topic-related	Textbook	Book chapter	Secondary
**123**	Hammond and Joynt Maddox, 2019	A Theoretical Framework for Clinical Implementation of Social Determinants of Health	Topic-related	Journal article	Perspective	Secondary
**124**	Health Canada, 2021	Building a National Strategy for High-Cost Drugs for Rare Diseases: A Discussion Paper for Engaging Canadians	Topic-related	Governmental document	Official discussion paper	Secondary
**125**	Healthy People 2030, 2023	Social Determinants of Health	Topic-related	Official website	Online briefing	Secondary
**126**	Hedley et al, 2023	Together4RD position statement on collaboration between European reference networks and industry	Topic-related	Journal article	Position statement	Secondary
**127**	Henderson et al, 2024	Gene Therapy for Genetic Syndromes: Understanding the Current State to Guide Future Care	Topic-related	Journal article	Literature review	Secondary
**128**	Henrard and Arickx, 2016	Negotiating prices of drugs for rare diseases	Topic-related	Journal article	Perspective	Secondary
**129**	Herder, 2017	What Is the Purpose of the Orphan Drug Act?	Topic-related	Journal article	Perspective	Secondary
**130**	Hivert et al, 2022	IRDiRC: 1000 new rare diseases treatments by 2027, identifying and bringing forward strategic actions	Topic-related	Journal article	Perspective	Secondary
**131**	Holme et al, 2024	Moving towards Normalization of haemostasis and health	Topic-related	Journal article	Literature review	Secondary

		equity: Evolving treatment goals for haemophilia A				
**132**	House of Commons of Canada, 2019	CANADIANS AFFECTED BY RARE DISEASES AND DISORDERS: IMPROVING ACCESS TO TREATMENT - Report of the Standing Committee on Health	Topic-related	Governmental document	Official report	Secondary
**133**	Hsu et al, 2018	Disease and economic burden for rare diseases in Taiwan: a longitudinal study using Taiwan's National Health Insurance Research Database	Topic-related	Journal article	Original research article	Primary
**134**	Huber et al, 2011	How should we define health?	Topic-related	Journal article	Perspective	Secondary
**135**	Hyde and Shortell, 2012	The Structure and Organization of Local and State Public Health Agencies in the U.S.: A Systematic Review	Topic-related	Journal article	Systematic review	Secondary
**136**	İNCE and GÜRE, 2021	Evaluation of rare diseases policy performance of oecd countries using mcdm methods	Topic-related	Journal article	Secondary analysis	Secondary
**137**	Incerti et al, 2022	Cost-effectiveness of genome sequencing for diagnosing patients with undiagnosed rare genetic diseases	Topic-related	Journal article	Secondary analysis	Secondary
**138**	Institute of Medicine, 2010	Rare diseases and orphan products: accelerating research and development	Topic-related	Textbook	Book	Secondary
**139**	Institute of Medicine, 2011	A Nationwide Framework for Surveillance of Cardiovascular and Chronic Lung Diseases	Topic-related	Textbook	Book chapter	Secondary

**140**	Iorio et al, 2019	Establishing the Prevalence and Prevalence at Birth of Hemophilia in Males: A Meta-analytic Approach Using National Registries	Topic-related	Journal article	Secondary analysis	Secondary
**141**	Islam et al, 2019	Social Determinants of Health and Related Inequalities: Confusion and Implications	Topic-related	Journal article	Perspective	Secondary
**142**	IQVIA, 2021	Innovative Funding Models for Treatment of Rare Diseases	Topic-related	Report	Overview	Secondary
**143**	Jakubowski et al, 2024	Clinical and non-clinical aspects of reimbursement policy for orphan drugs in selected European countries	Topic-related	Journal article	Original research article	Primary
**144**	Jommi et al, 2024	Price and reimbursement for orphan medicines and managed entry agreements: does Italy need a framework?	Topic-related	Journal article	Perspective	Secondary
**145**	Kaczmarek et al, 2024	Current and emerging gene therapies for haemophilia A and B	Topic-related	Journal article	Overview	Secondary
**146**	Karkee, 2014	Public Health Education in South Asia: A Basis for Structuring a Master Degree Course	Topic-related	Journal article	Perspective	Secondary
**147**	van Karnebeek et al, 2022	Leaving no patient behind! Expert recommendation in the use of innovative technologies for diagnosing rare diseases	Topic-related	Journal article	Recommen dations	Secondary
**148**	Kaywanga et al, 2022	Rare diseases in Tanzania: a National Call for Action to address policy and urgent needs of individuals with rare diseases	Topic-related	Journal article	Position statement	Secondary

**149**	Kenny and Stone, 2022	Psychological support at diagnosis of a rare disease	Topic-related	Journal article	Literature review	Secondary
**150**	Khosla and Valdez, 2018	A compilation of national plans, policies and government actions for rare diseases in 23 countries	Topic-related	Journal article	Literature review	Secondary
**151**	Kim, 2013	The Orphan Drug Act: How the FDA Unlawfully Usurped Market Exclusivity	Topic-related	Journal article	Perspective	Secondary
**152**	Kingdom and Wright, 2022	Incomplete Penetrance and Variable Expressivity: From Clinical Studies to Population Cohorts	Topic-related	Journal article	Literature review	Secondary
**153**	Kisling and Das, 2023	Prevention strategies	Topic-related	Textbook	Book chapter	Secondary
**154**	Kloosterman et al, 2022	The bleeding phenotype in people with nonsevere hemophilia	Topic-related	Journal article	Original research article	Primary
**155**	Knowledge Ecology International, 2023	Orphan Drug Timeline	Topic-related	Professional website	Online briefing	Secondary
**156**	Kohn et al, 2023	Successes and challenges in clinical gene therapy	Topic-related	Journal article	Overview	Secondary
**157**	Krahn et al, 2021	It's time to reconsider how we define health: Perspective from disability and chronic condition	Topic-related	Journal article	Commentary	Secondary
**158**	Kraus et al, 2022	Literature reviews as independent studies: guidelines for academic practice	Methodological	Journal article	Overview	Secondary

**159**	Lane et al, 2016	Understanding stakeholder important outcomes and perceptions of equity, acceptability and feasibility of a care model for haemophilia management in the US: a qualitative study	Topic-related	Journal article	Original research article	Primary
**160**	Lavandeira, 2002	Orphan drugs: legal aspects, current situation	Topic-related	Journal article	Overview	Secondary
**161**	Lee, 2021	Pricing and Reimbursement Pathways of New Orphan Drugs in South Korea: A Longitudinal Comparison	Topic-related	Journal article	Literature review	Secondary
**162**	Leonardi, 2018	The Definition of Health: Towards New Perspectives	Topic-related	Journal article	Perspective	Secondary
**163**	Li et al, 2022	Rare disease curative care expenditure-financing scheme-health provider–beneficiary group analysis: an empirical study in Sichuan Province, China	Topic-related	Journal article	Original research article	Primary
**164**	Li et al, 2023	Policy analysis in the field of rare diseases in China: a combined study of content analysis and Bibliometrics analysis	Topic-related	Journal article	Literature review	Secondary
**165**	Lim et al, 2019	The cumulative incidence and trends of rare diseases in South Korea: a nationwide study of the administrative data from the National Health Insurance Service database from 2011-2015	Topic-related	Journal article	Original research article	Primary
**166**	Liu et al, 2023	Long way to go: Progress of orphan drug accessibility in China from 2017 to 2022	Topic-related	Journal article	Original research article	Primary

**167**	Loeber et al, 2021	Neonatal Screening in Europe Revisited: An ISNS Perspective on the Current State and Developments Since 2010	Topic-related	Journal article	Perspective	Secondary
**168**	Lopes-Júnior et al, 2022	Health Policies for Rare Disease Patients: A Scoping Review	Topic-related	Journal article	Scoping review	Secondary
**169**	Lopes-Bastida et al, 2016	Social/economic costs and health-related quality of life in patients with rare diseases in Europe	Topic-related	Journal article	Editorial	Secondary
**170**	Lorey, 2017	Genetic Determinants of Disease and Genetics in Public Health	Topic-related	Textbook	Book chapter	Secondary
**171**	Lu and Han, 2022	The definition of rare disease in China and its prospects	Topic-related	Journal article	Commentar y	Secondary
**172**	De Luca and Cossu, 2023	Cost and availability of novel cell and gene therapies	Topic-related	Journal article	Perspective	Secondary
**173**	Magalhaes, 2022	Should rare diseases get special treatment?	Topic-related	Journal article	Perspective	Secondary
**174**	Mager, 2019	Overview of Community, Public, and Population Health	Topic-related	Textbook	Book chapter	Secondary
**175**	Mahlangu et al, 2024	Diagnosis and treatment challenges in lower resource countries: State-of-the-art	Topic-related	Journal article	Literature review	Secondary
**176**	Makarova et al, 2021	Evaluation of orphan diseases global burden	Topic-related	Journal article	Overview	Secondary
**177**	Makris et al, 2011	EUHASS: The European Haemophilia Safety Surveillance System	Topic-related	Journal article	Overview	Secondary

**178**	Malec et al, 2023	Targeting higher factor VIII levels for prophylaxis in haemophilia A: a narrative review	Topic-related	Journal article	Literature review	Secondary
**179**	Malhan et al, 2021	Cost of hemophilia A in Turkey: an economic disease burden analysis.	Topic-related	Journal article	Secondary analysis	Secondary
**180**	Manco-Johnson et al, 2018	Community counts: Evolution of a national surveillance system for bleeding disorders	Topic-related	Journal article	Letter to the Editor	Secondary
**181**	Marinello et al, 2022	Empowering rare disease patients through patient education: the new BehçeTalk programme	Topic-related	Journal article	Commentar y	Secondary
**182**	Marmot et al, 2008	Closing the gap in a generation: health equity through action on the social determinants of health	Topic-related	Journal article	Perspective	Secondary
**183**	Marmot and Bell, 2012	Fair society, healthy lives	Topic-related	Journal article	Overview	Secondary
**184**	Marolt Presen et al, 2025	CTGCT, Centre of Excellence for the Technologies of Gene and Cell Therapy: Collaborative translation of scientific discoveries into advanced treatments for neurological rare genetic diseases and cancer	Topic-related	Journal article	Overview	Secondary
**185**	Mayrides et al, 2020	A civil society view of rare disease public policy in six Latin American countries	Topic-related	Journal article	Literature review	Secondary
**186**	Mazzucato et al, 2014	A population-based registry as a source of health indicators for rare diseases: the ten-year experience of	Topic-related	Journal article	Original research article	Primary

		the Veneto Region's rare diseases registry				
**187**	McCartney et al, 2019	Defining health and health inequalities	Topic-related	Journal article	Literature review	Secondary
**188**	Mehta and Reddivari, 2022	Hemophilia	Topic-related	Textbook	Book chapter	Secondary
**189**	Mikami, 2019	Orphans in the Market: The History of Orphan Drug Policy	Topic-related	Journal article	Literature review	Secondary
**190**	Miller et al, 2022	Occurrence rates of inherited bleeding disorders other than haemophilia and von Willebrand disease among people receiving care in specialized treatment centres in the United States	Topic-related	Journal article	Letter to the Editor	Secondary
**191**	Mincarone et al, 2017	Reimbursed price of orphan drugs: current strategies and potential improvements	Topic-related	Journal article	Overview	Secondary
**192**	Miyamoto and Kakkis, 2011	The potential investment impact of improved access to accelerated approval on the development of treatments for low prevalence rare diseases	Topic-related	Journal article	Literature review	Secondary
**193**	Modell et al, 2018	Congenital disorders: epidemiological methods for answering calls for action	Topic-related	Journal article	Editorial	Secondary
**194**	Moliner and Waligora, 2017	The European Union Policy in the Field of Rare Diseases	Topic-related	Textbook	Book chapter	Secondary

**195**	Monaco et al, 2022	Research on rare diseases: ten years of progress and challenges at IRDiRC	Topic-related	Journal article	Overview	Secondary
**196**	Moutselos and Maglogiannis, 2020	Evidence-based Public Health Policy Models Development and Evaluation using Big Data Analytics and Web Technologies	Topic-related	Journal article	Overview	Secondary
**197**	Mu et al, 2022	A Cross-Sectional Study of Price and Affordability of Drugs for Rare Diseases in Shandong Province, China	Topic-related	Journal article	Original research article	Primary
**198**	Mukherjee, 2019	Care for Rare: Spotlight on Rare Diseases	Topic-related	Journal article	Editorial	Secondary
**199**	Nabbout et al, 2023	Towards the international interoperability of clinical research networks for rare diseases: recommendations from the IRDiRC Task Force	Topic-related	Journal article	Position statement	Secondary
**200**	Nacul et al, 2014	A Toolkit to assess health needs for congenital disorders in low- and middle-income countries: an instrument for public health action	Topic-related	Journal article	Overview	Secondary
**201**	National Centre for Advancing Translational Sciences, 2024	Bespoke Gene Therapy Consortium (BGTC)	Topic-related	Official website	Online briefing	Secondary
**202**	National Institutes of Health, 2021	NIH, FDA and 15 private organizations join forces to increase effective gene therapies for rare diseases	Topic-related	Official website	Online briefing	Secondary
**203**	Naumova, 2022	Precision public health: is it all about the data?	Topic-related	Journal article	Editorial	Secondary

**204**	Ndoumba-Mintya et al, 2023	Optimizing Haemophilia Care in Resource-Limited Countries: Current Challenges and Future Prospects	Topic-related	Journal article	Literature review	Secondary
**205**	Nguengang Wakap et al, 2020	Estimating cumulative point prevalence of rare diseases: analysis of the Orphanet database	Topic-related	Journal article	Original research article	Primary
**206**	Ninomiya and Okura, 2022	Nationwide comprehensive epidemiological study of rare diseases in Japan using a health insurance claims database	Topic-related	Journal article	Original research article	Primary
**207**	Nolte et al, 2019	A Systematic Review of Orthopedic Global Outreach Efforts Based on WHO-CHOICE Thresholds	Topic-related	Journal article	Systematic review	Secondary
**208**	Nori et al, 2023	The global role of patients, advocates and caregivers in rare diseases	Topic-related	Journal article	Literature review	Secondary
**209**	Nsubuga et al, 2006	Public health surveillance: a tool for targeting and monitoring interventions	Topic-related	Textbook	Book chapter	Secondary
**210**	O'Connor, 2013	Orphan drug designation–Europe, the USA and Japan	Topic-related	Journal article	Editorial	Secondary
**211**	O'Hara et al, 2017	The cost of severe haemophilia in Europe: the CHESS study	Topic-related	Journal article	Original research article	Primary
**212**	Okolo et al, 2019	Population-based surveillance of haemophilia and patient outcomes in Indiana using multiple data sources	Topic-related	Journal article	Original research article	Primary
**213**	De Oliveira et al, 2024	Epidemiological characterization of rare diseases in Brazil: A retrospective study of the	Topic-related	Journal article	Original research article	Primary

		Brazilian Rare Diseases Network				
**214**	Ollendorf et al, 2018	Evaluating and Valuing Drugs for Rare Conditions: No Easy Answers	Topic-related	Journal article	Literature review	Secondary
**215**	Orphanet, 2023	Orphan drugs in Japan	Topic-related	Official website	Online briefing	Secondary
**216**	Orphanet, 2023	Orphan drugs in the United States of America	Topic-related	Official website	Online briefing	Secondary
**217**	Ou et al, 2024	Artificial intelligence empowering rare diseases: a bibliometric perspective over the last two decades	Topic-related	Journal article	Literature review	Secondary
**218**	Pai et al, 2016	NHF-McMaster guideline on care models for haemophilia management	Topic-related	Journal article	Systematic review	Secondary
**219**	Palagyi et al, 2024	Systematic scoping review of socioeconomic burden and associated psychosocial impact in patients with rare kidney diseases and their caregivers	Topic-related	Journal article	Systematic review	Secondary
**220**	Park et al, 2019	Systematic review of basket trials, umbrella trials, and platform trials: a landscape analysis of master protocols	Topic-related	Journal article	Systematic review	Secondary
**221**	Parker et al, 2014	Meeting the emerging public health needs of persons with blood disorders	Topic-related	Journal article	Literature review	Secondary
**222**	Parliament of the Commonwealth of Australia, 2021	The New Frontier - Delivering better health for all Australians - Inquiry into approval processes for new drugs and novel medical technologies in Australia	Topic-related	Official document	Official report	Secondary

**223**	Partin, 2022	Rarely mentioned: how we arrived at the quantitative definition of a rare disease	Topic-related	Journal article	Perspective	Secondary
**224**	Patwardhan et al, 2015	Concepts of Health and Disease	Topic-related	Textbook	Book chapter	Secondary
**225**	Pavan et al, 2017	Clinical practice guidelines for rare diseases: the orphanet database	Topic-related	Journal article	Literature review	Secondary
**226**	Pearson et al, 2018	Economic Modeling Considerations for Rare Diseases	Topic-related	Journal article	Literature review	Secondary
**227**	Pearson et al, 2022	The next generation of rare disease drug policy: ensuring both innovation and affordability	Topic-related	Journal article	Literature review	Secondary
**228**	Perolla and Kalaja, 2024	Improving Hemophilia Care in Low- and Middle-Income Countries: Addressing Challenges and Enhancing Quality of Life	Topic-related	Journal article	Literature review	Secondary
**229**	Peyvandi et al, 2020	Kreuth V initiative: European consensus proposals for treatment of hemophilia using standard products, extended half-life coagulation factor concentrates and non-replacement therapies	Topic-related	Journal article	Recommen dations	Secondary
**230**	Pierce et al, 2022	Achieving access to haemophilia care in low-income and lower-middle-income countries: expanded Humanitarian Aid Program of the World Federation of Hemophilia after 5 years	Topic-related	Journal article	Overview	Secondary
**231**	Postma et al, 2022	Assessing the value of orphan drugs using conventional cost-	Topic-related	Journal article	Literature review	Secondary

		effectiveness analysis: Is it fit for purpose?				
**232**	Qamar et al, 2023	The role of medical students in advocacy for rare diseases – Experience from a Low- and Middle-Income Country (LMIC)	Topic-related	Journal article	Perspective	Secondary
**233**	Rahit, and Tarailo-Graovac, 2020	Genetic Modifiers and Rare Mendelian Disease	Topic-related	Journal article	Literature review	Secondary
**234**	Rajueni and Chakraborty Choudhury, 2023	Assessment of the availability of repurposed orphan drugs in India	Topic-related	Journal article	Original research article	Primary
**235**	Raphael et al, 2020	Social determinants of health: The Canadian facts	Topic-related	Textbook	Book	Secondary
**236**	Rejtő et al, 2023	Bleeding phenotype in nonsevere hemophilia by International Society on Thrombosis and Haemostasis bleeding assessment tool, bleeding frequency, and the joint status	Topic-related	Journal article	Original research article	Primary
**237**	Richardson, et al, 2024	Impact of mental health in persons living with rare disease: Findings from the AMCP Market Insights Program	Topic-related	Journal article	Overview	Secondary
**238**	Richter et al, 2015	Rare Disease Terminology and Definitions-A Systematic Global Review: Report of the ISPOR Rare Disease Special Interest Group	Topic-related	Journal article	Systematic review	Secondary

**239**	Rihm, et al, 2022	The psychosocial situation of families caring for children with rare diseases during the COVID-19 pandemic: results of a cross-sectional online survey	Topic-related	Journal article	Original research article	Primary
**240**	Rodwell and Aymé, 2015	Rare disease policies to improve care for patients in Europe	Topic-related	Journal article	Literature review	Secondary
**241**	Rollet et al, 2013	Sustainable rare diseases business and drug access: no time for misconceptions	Topic-related	Journal article	Literature review	Secondary
**242**	Roux, 2016	On the Distinction--or Lack of Distinction--Between Population Health and Public Health	Topic-related	Journal article	Perspective	Secondary
**243**	Rubin et al, 2022	Limitations of standard cost-effectiveness methods for health technology assessment of treatments for rare, chronic diseases: a case study of treatment for cystic fibrosis	Topic-related	Journal article	Secondary analysis	Secondary
**244**	Saimbert, 2017	Key Principles for Searching the Literature	Methodological	Textbook	Book chapter	Secondary
**245**	Sakushima et al, 2021	Orphan drug designation and development in Japan: 25 years of experience and assessment	Topic-related	Journal article	Overview	Secondary
**246**	Salzman et al, 2018	Addressing the Value of Gene Therapy and Enhancing Patient Access to Transformative Treatments	Topic-related	Journal article	Position statement	Secondary
**247**	De Santis et al, 2019	Patient empowerment of people living with rare diseases. Its contribution to	Topic-related	Journal article	Literature review	Secondary

		sustainable and resilient healthcare systems.				
**248**	Schieve et al, 2020	Evaluation of CDC's Hemophilia Surveillance Program - Universal Data Collection (1998-2011) and Community Counts (2011-2019), United States	Topic-related	Journal article	Overview	Secondary
**249**	Schouten et al, 2020	Selected government definitions of orphan or rare diseases - KEI briefing note 2020:4	Topic-related	Briefing note	Overview	Secondary
**250**	Shah, 2006	Regulatory framework for the treatment of orphan diseases	Topic-related	Textbook	Book chapter	Secondary
**251**	Sharma et al, 2022	Ophthalmic registries for rare eye diseases	Topic-related	Journal article	Systematic review	Secondary
**252**	Sheng, 2023	Connecting Each Other in Rare Diseases: A Call for Cross-Regional Collaboration	Topic-related	Textbook	Book chapter	Secondary
**253**	Simoens, 2011	Pricing and reimbursement of orphan drugs: the need for more transparency	Topic-related	Journal article	Literature review	Secondary
**254**	Skinner et al, 2020	Achieving the unimaginable: health equity in haemophilia	Topic-related	Journal article	Literature review	Secondary
**255**	Solar and Irwin, 2010	A conceptual framework for action on the Social Determinants of Health Discussion Paper 2 (Policy and Practice)	Topic-related	Official document	Official discussion paper	Secondary
**256**	Song et al, 2012	Rare diseases, orphan drugs, and their regulation in Asia: Current status and future perspectives	Topic-related	Journal article	Literature review	Secondary

**257**	Song et al, 2013	Rare diseases and orphan drugs in Japan: developing multiple strategies of regulation and research	Topic-related	Journal article	Editorial	Secondary
**258**	Song et al, 2021	Rare Neurovascular Diseases in Korea: Classification and Related Genetic Variants	Topic-related	Journal article	Literature review	Secondary
**259**	Soucie, 2012	Public health surveillance and data collection: general principles and impact on hemophilia care	Topic-related	Journal article	Perspective	Secondary
**260**	Soucie, 2014a	A Public Health Approach to the Prevention of Inhibitors in Hemophilia	Topic-related	Journal article	Overview	Secondary
**261**	Soucie et al, 2014b	A study of prospective surveillance for inhibitors among persons with haemophilia in the United States	Topic-related	Journal article	Original research article	Primary
**262**	Soucie et al, 2014c	National surveillance for hemophilia inhibitors in the United States: Summary report of an expert meeting	Topic-related	Journal article	Perspective	Secondary
**263**	Spronk et al, 2019	Calculating incidence rates and prevalence proportions: not as simple as it seems	Topic-related	Journal article	Original research article	Primary
**264**	Srivastava et al, 2020	WFH guidelines for the management of hemophilia	Topic-related	Journal article	Practice guidelines	Secondary
**265**	Srivastava, 2021	The case for equitable haemophilia care	Topic-related	Journal article	Letter	Secondary
**266**	Stonebraker et al, 2020	The World Federation of Hemophilia Annual Global Survey 1999-2018	Topic-related	Journal article	Overview	Secondary

**267**	Svalastog et al, 2017	Concepts and definitions of health and health-related values in the knowledge landscapes of the digital society	Topic-related	Journal article	Overview	Secondary
**268**	Takemura and Sone, 2019	Research and development on intractable & rare diseases in Japan: Contribution of the National Institute of Public Health to research program management	Topic-related	Journal article	Literature review	Secondary
**269**	Taruscio et al, 2014	European Recommendations for Primary Prevention of Congenital Anomalies	Topic-related	Journal article	Practice guidelines	Secondary
**270**	Taruscio, 2022	The long journey of people with rare diseases: from darkness to the UN Resolution 2022	Topic-related	Journal article	Editorial	Secondary
**271**	Thomson et al, 2014	BASIC LITERATURE SEARCH STRATEGIES	Methodological	Textbook	Book chapter	Secondary
**272**	Thomson et al, 2018	The effects of public health policies on health inequalities in high-income countries: an umbrella review	Topic-related	Journal article	Systematic review	Secondary
**273**	Thorpe et al, 2008	The three domains of public health: An internationally relevant basis for public health education?	Topic-related	Journal article	Literature review	Secondary
**274**	Tisdale et al, 2021	The IDeaS initiative: pilot study to assess the impact of rare diseases on patients and healthcare systems	Topic-related	Journal article	Original research article	Primary
**275**	Tran et al, 2023	Building the foundation for a community-generated national research blueprint for inherited bleeding	Topic-related	Journal article	Perspective	Secondary

		disorders: research priorities to transform the care of people with hemophilia				
**276**	Tran et al, 2024	The importance and evolution of bleeding disorder registries	Topic-related	Journal article	Literature review	Secondary
**277**	Tsai et al, 2015	Cost-effectiveness analysis of carrier and prenatal genetic testing for X-linked hemophilia	Topic-related	Journal article	Original research article	Primary
**278**	Tumienė et al, 2024	Rare diseases: still on the fringes of universal health coverage in Europe	Topic-related	Journal article	Perspective	Secondary
**279**	U.S. Congress, 1983	Orphan Drug Act	Topic-related	Official document	Legislation	Primary
**280**	U.S. Department of Health and Human Services, 2001	The Orphan Drug Act: Implementation and Impact	Topic-related	Official document	Official report	Primary
**281**	U.S. Department of Health and Human Services, 2019	Expedited Programs for Regenerative Medicine Therapies for Serious Conditions - Guidance for Industry	Topic-related	Official document	Guidance	Primary
**282**	U.S. FDA, 2022	CDER Continues to Make Rare Diseases a Priority with Drug Approvals and Programming to Speed Therapeutic Development	Topic-related	Official website	Online briefing	Secondary
**283**	Udompap et al, 2015	Current and Future Burden of Chronic Nonmalignant Liver Disease	Topic-related	Journal article	Literature review	Secondary

**284**	Uhlenbusch et al, 2019	Perceived burden in dealing with different rare diseases: a qualitative focus group study	Topic-related	Journal article	Original research article	Primary
**285**	United Nations, 2015	Transforming our world : the 2030 Agenda for Sustainable Development : resolution 70/1 / adopted by the General Assembly on 25 September 2015	Topic-related	Official document	Official resolution	Secondary
**286**	United Nations, 2019	Political Declaration of the High-Level Plenary Meeting on Universal Health Coverage :resolution 74/2 / adopted by the General Assembly on 10 October 2019	Topic-related	Official document	Official resolution	Secondary
**287**	United Nations, 2021	Addressing the challenges of persons living with a rare disease and their families : resolution 76/132 / adopted by the General Assembly on 16 December 2021	Topic-related	Official document	Official resolution	Secondary
**288**	Valdez et al, 2016	Public Health and Rare Diseases: Oxymoron No More	Topic-related	Journal article	Perspective	Secondary
**289**	Valdez et al, 2017	The need for a next-generation public health response to rare diseases	Topic-related	Journal article	Commentar y	Secondary
**290**	Valentino et al, 2021	Integrated Hemophilia Patient Care via a National Network of Care Centers in the United States: A Model for Rare Coagulation Disorders	Topic-related	Journal article	Perspective	Secondary
**291**	Velmovitsk y et al, 2021	Convergence of Precision Medicine and Public Health Into Precision Public Health	Topic-related	Journal article	Literature review	Secondary

**292**	Verma et al, 2022	Transformative effect of a Humanitarian Program for individuals affected by rare diseases: building support systems and creating local expertise	Topic-related	Journal article	Literature review	Secondary
**293**	Villalón-García et al, 2020	Precision Medicine in Rare Diseases	Topic-related	Journal article	Literature review	Secondary
**294**	van Vulpen et al, 2017	The current state of adverse event reporting in hemophilia	Topic-related	Journal article	Literature review	Secondary
**295**	Wainstock and Katz, 2023	Advancing rare disease policy in Latin America: a call to action	Topic-related	Journal article	Perspective	Secondary
**296**	Ward, 2013	Estimating disease prevalence and incidence using administrative data: some assembly required	Topic-related	Journal article	Editorial	Secondary
**297**	Wenzl and Chapman, 2019	Performance-based managed entry agreements for new medicines in OECD countries and EU member states: How they work and possible improvements going forward	Topic-related	Official document	Official report	Secondary
**298**	Wilkinson and Marmot, 2003	Social determinants of health: the solid facts	Topic-related	Textbook	Book	Secondary
**299**	WFH, 2024	Report on the Annual Global Survey 2023	Topic-related	Professional website	Online briefing	Secondary
**300**	WHO, 1946	Constitution of the World Health Organization	Topic-related	Official document	Guidance	Primary

**301**	WHO, 2017	Determinants of health	Topic-related	Official website	Online briefing	Secondary
**302**	WHO, 2023	Congenital disorders	Topic-related	Official website	Online briefing	Secondary
**303**	WHO, 2024	Social determinants of health	Topic-related	Official website	Online briefing	Secondary
**304**	WHO Regional Office for Africa, 2023	Overview (Public health surveillance)	Topic-related	Official website	Online briefing	Secondary
**305**	WHO Regional Office for the Eastern Mediterranean Region, 2017	Assessment of essential public health functions in countries of the Eastern Mediterranean Region: assessment tool	Topic-related	Official document	Official report	Secondary
**306**	Witt et al, 2023	Living with a rare disease - experiences and needs in pediatric patients and their parents	Topic-related	Journal article	Original research article	Primary
**307**	Worldometer, n.d.	Japan Population	Topic-related	Professional website	Online briefing	Secondary
**308**	Yang et al, 2022	The national economic burden of rare disease in the United States in 2019	Topic-related	Journal article	Original research article	Primary
**309**	Yang et al, 2024	Biomedical Big Data Technologies, Applications, and Challenges for Precision Medicine: A Review	Topic-related	Journal article	Literature review	Secondary
**310**	Yates and Hinkel, 2022	The economics of moonshots: Value in rare disease drug development	Topic-related	Journal article	Perspective	Secondary

**311**	Ying et al, 2021	An update on China's national policies regarding rare diseases	Topic-related	Journal article	Literature review	Secondary
**312**	Yoon et al, 2023	Prioritization of research engaged with rare disease stakeholders: a systematic review and thematic analysis	Topic-related	Journal article	Systematic review	Secondary
**313**	Zanello et al, 2022	Recommendations from the IRDiRC Working Group on methodologies to assess the impact of diagnoses and therapies on rare disease patients	Topic-related	Journal article	Position statement	Secondary
**314**	Zhou et al, 2022	Influencing Factors of Health Technology Assessment to Orphan Drugs: Empirical Evidence in England, Scotland, Canada, and Australia	Topic-related	Journal article	Literature review	Secondary
**315**	Zhou et al, 2022	Burden of illness: direct and indirect costs among persons with hemophilia A in the United States	Topic-related	Journal article	Original research article	Primary

Supplementary Table 2. Social determinants of health and their main categories (1–8).

Ser.	Determinant of health	Category
1	Access to affordable and quality healthcare services	Social and economic environment
2	Access to green space	Physical environment
3	Access to the internet and information	Social and economic environment
4	Addiction	Personal characteristics and behaviors
5	Bill payment assistance	Social and economic environment
6	Civil participation	Social and economic environment
7	Climate change	Physical environment
8	Colonialism and marginalization	Physical environment
9	Community engagement	Social and economic environment
10	Contraceptive use	Personal characteristics and behaviors
11	Crime and violence	Physical environment
12	Culture or social norms	Social and economic environment
13	Data and surveillance	Social and economic environment
14	Depression	Personal characteristics and behaviors
15	Development agendas	Social and economic environment
16	Early childhood development	Personal characteristics and behaviors
17	Economic stability	Social and economic environment
18	Education access and quality	Social and economic environment
19	Environmental conditions	Physical environment

**20**	Evaluation and evidence-building	Social and economic environment
**21**	Family	Social and economic environment
**22**	Financial stability	Social and economic environment
**23**	Food insecurity	Social and economic environment
**24**	Gender	Personal characteristics and behaviors
**25**	Health literacy	Personal characteristics and behaviors
**26**	Immigration	Physical environment
**27**	Income, its distribution, and social protection	Social and economic environment
**28**	Incarceration	Physical environment
**29**	Individual's legal status	Personal characteristics and behaviors
**30**	Infrastructure and capacity	Physical environment
**31**	Insurance status	Social and economic environment
**32**	Language and literacy	Personal characteristics and behaviors
**33**	Media	Social and economic environment
**34**	Oral health	Personal characteristics and behaviors
**35**	Partnerships and collaboration	Social and economic environment
**36**	Policy and law	Social and economic environment
**37**	Political systems	Social and economic environment
**38**	Poverty	Personal characteristics and behaviors
**39**	Quality of housing and living conditions	Physical environment
**40**	Race/ethnicity	Personal characteristics and behaviors

**41**	Religion	Personal characteristics and behaviors
**42**	Sexual orientation	Personal characteristics and behaviors
**43**	Social norms and community context	Social and economic environment
**44**	Social inclusion and non-discrimination	Social and economic environment
**45**	Structural conflict	Physical environment
**46**	Stress	Personal characteristics and behaviors
**47**	Time	Personal characteristics and behaviors
**48**	Transport system	Physical environment
**49**	Unemployment and job insecurity	Social and economic environment
**50**	Work conditions	Physical environment

References:

1. CDC. Social Determinants of Health at CDC [Internet]. 2022 [cited 2023 Sep 2]. Available from: https://www.cdc.gov/about/sdoh/index.html

2. Hammond G, Joynt Maddox KE. A Theoretical Framework for Clinical Implementation of Social Determinants of Health. JAMA Cardiol. 2019 Dec;4(12):1189–90.

3. Healthy People 2030. Social Determinants of Health [Internet]. [cited 2024 Apr 26]. Available from: https://health.gov/healthypeople/priority-areas/social-determinants-health

4. Islam MM. Social Determinants of Health and Related Inequalities: Confusion and Implications. Front public Heal. 2019;7:11.

5. Raphael D, Bryant T, Mikkonen J, Alexander R. Social determinants of health: The Canadian facts [Internet]. 2nd ed. Oshawa: Ontario Tech University Faculty of Health Sciences and Toronto: York University School of Health Policy and Management; 2020. 1–93 p. Available from: http://www.thecanadianfacts.org/

6. World Health Organization. Determinants of health [Internet]. 2017 [cited 2023 Sep 2]. Available from: https://www.who.int/news-room/questions-and-answers/item/determinants-of-health

7. World Health Organization. Social determinants of health [Internet]. 2024 [cited 2023 Sep 2]. Available from: https://www.who.int/health-topics/social-determinants-of-health#tab=tab_1

8. World Health Organization. Regional Office for the Eastern Mediterranean Region. Assessment of essential public health functions in countries of the Eastern Mediterranean Region: assessment tool [Internet]. Cairo; 2017. Available from: https://www.emro.who.int/about-who/public-health-functions/index.html
